# ISWI/CHRAC orchestrates sequential chromatin-remodeling activities by mobilizing DNA polymerase ε for homologous recombination

**DOI:** 10.1038/s42003-026-10862-0

**Published:** 2026-09-15

**Authors:** Jianghao Qian, Kozo Tanaka, Akira Yasui, Ayako Ui

**Affiliations:** 1 Department of Molecular Oncology, Institute of Development, Aging and Cancer (IDAC), Tohoku University, Sendai, Japan; 2 IDAC Fellow Laboratory, Institute of Development, Aging and Cancer (IDAC), Tohoku University, Sendai, Japan

**Keywords:** DNA recombination, Chromatin remodelling

## Abstract

ATP-dependent chromatin remodeling is essential for replication, transcription, and DNA repair, especially DNA double-strand break (DSB) repair. However, the mechanisms underlying chromatin remodeling remain elusive. Here, we investigated the role of CHRAC17, a component of the chromatin assembly complex (CHRAC), in maintaining genome stability. In response to DSBs, the ISWI-family ATPase complex SNF2H-ACF1 is immediately recruited to DSBs through the H2B-type histone-fold protein CHRAC17, forming CHRAC, which promotes nucleosome assembly and recruits BRCA1 and RAD51 to the DSB site. CHRAC promotes later RAD51-mediated HR steps through additional nucleosome-remodeling activity, including nucleosome sliding activity. CHRAC17, also known as POLE3, is a subunit of DNA polymerase ε (Polε). Polε is recruited to DSBs via CHRAC17, facilitating RAD51-mediated homologous recombination (HR) and conferring resistance to PARP inhibitors. These findings define a mechanism of homologous recombination driven by coordinated regulation of nucleosome dynamics by CHRAC and Polε, thereby ensuring genome stability.

**Figure:**
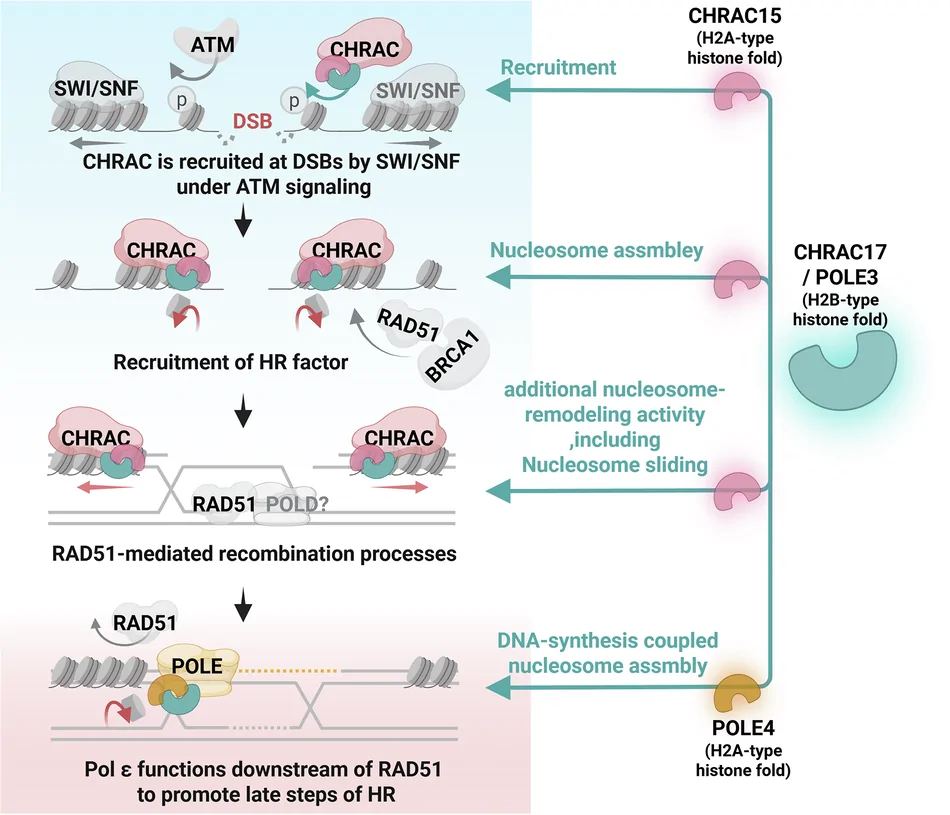

## Abstract (web summary)

CHRAC17, a key coordinator of chromatin remoder and DNA polymerase ε (Polε), orchestrates sequential chromatin remodeling, nucleosome assembly and sliding, to promote RAD51-mediated HR and mobilize Polε, thereby maintaining genome stability and conferring resistance to PARP inhibitors.

## Introduction

Adenosine triphosphate (ATP)-dependent chromatin remodeling (nucleosome remodeling) complexes regulate and control nucleosome positioning for transcription, DNA replication, and DNA repair. These complexes are categorized into the switch/sucrose non-fermentable (SWI/SNF), imitation switch (ISWI), INO80, and chromatin helicase DNA-binding (CHD) families according to the specific ATPase present^1^. Recent studies have reported various cancer cells to exhibit extremely high frequencies of mutations in the genes involved in chromatin remodeling^2,3^. The tumor-suppressive activity of remodeling factors can be attributed to their role in transcriptional regulation^1^. Furthermore, we and other groups have reported that the SWI/SNF- and ISWI-family chromatin-remodeling complexes are essential for non-homologous end joining (NHEJ) and homologous recombination (HR) in the occurrence of DNA double-strand break (DSB) repair, and this involvement in DSB repair has also been suggested to contribute to tumor suppression^2,4–8^.

Five different ISWI-family complexes harboring SNF2H as the catalytic subunit have been identified in human cells: ATP-dependent chromatin assembly and remodeling factor (ACF), nucleolar remodeling complex (NoRC), RSF, WSTF-ISWI chromatin-remodeling complex (WICH), and chromatin assembly complex (CHRAC)^9^. The ISWI-family complexes remodel chromatin in order to control various cellular processes including transcription, DNA replication, and DNA repair^1^. It is unclear how ISWI moves nucleosomes during cellular processes, including for DNA repair, although two different chromatin-remodeling activities have been detected for this complex in vitro; namely, nucleosome assembly and nucleosome sliding^1,9–11^. However, the remodeling activities required for each cellular function, including DSB repair, are poorly understood.

One protein of the CHRAC family, CHRAC17 (an H2B-type histone-fold protein) is also included the DNA polymerase ε complex (Pol ε) family, with the alternative name of POLE3. In vitro, CHRAC17 has been shown to form a complex with CHRAC15—an H2A-type histone-fold protein—to form an H2A/H2B-type histone-fold protein complex, which promotes the nucleosome-binding activity and remodeling activities of ACF1-SNF2H^10,11^. In Polε, CHRAC17 forms a histone chaperone complex with POLE4, an H2A-type histone-fold protein, which then combines with POLE1 and POLE2 to form the Pol ε complex^12,13^. It remains unclear whether CHRAC17 coordinates the functions of CHRAC and Pol ε in the same cellular processes, including DSB repair. Furthermore, the role of Pol ε in DSB repair remains elusive in humans and is poorly understood even in yeast^12^. This study elucidates the mechanism of HR—which plays an important role in genome stability—by evaluating the role of CHRAC and the Pol ε complex in DSB repair.

## Results

### CHRAC accumulates at DNA DSB sites via CHRAC17

**Fig. 1:**
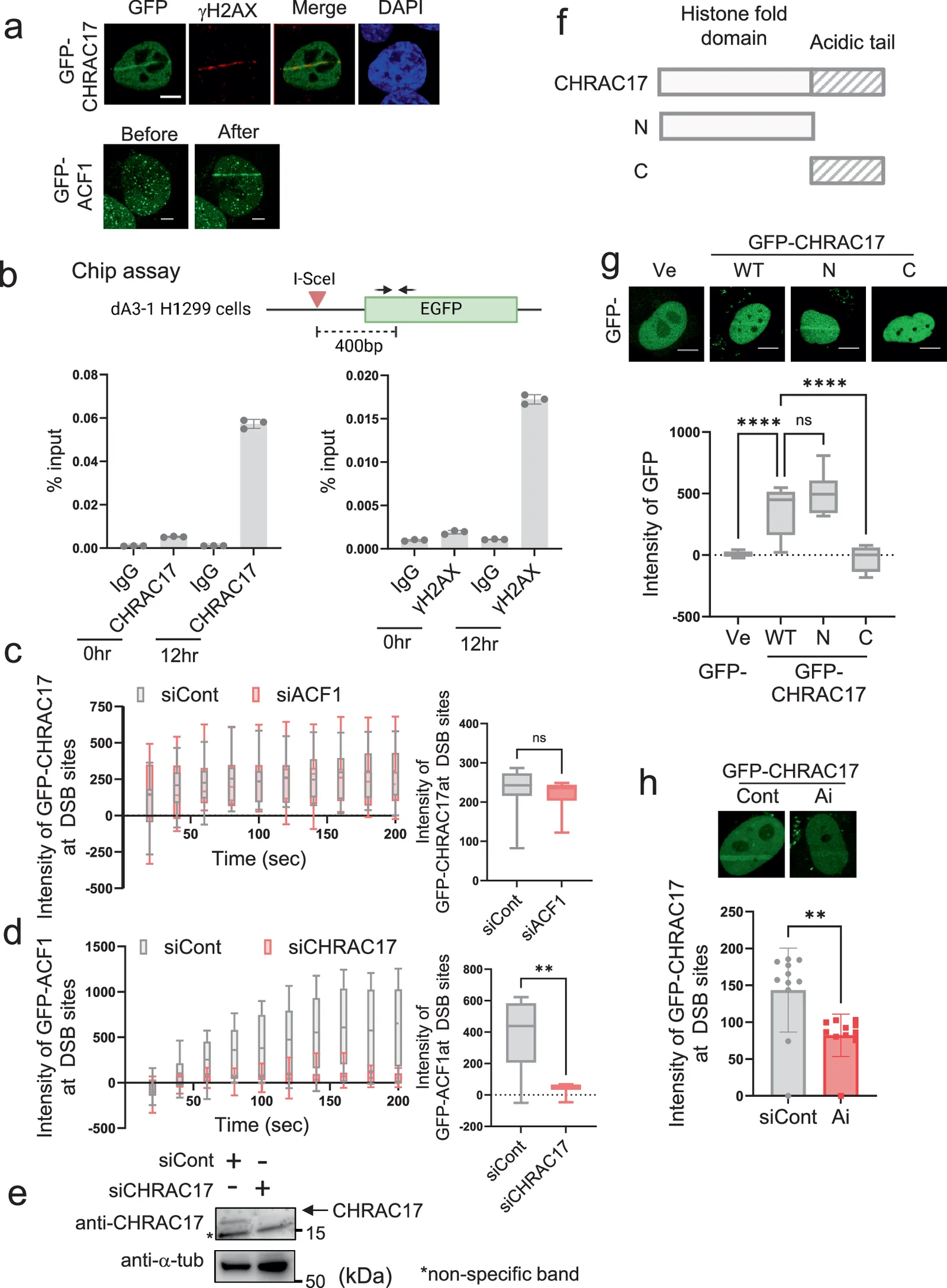
Chromatin assembly complex is recruited to DNA double-strand break sites via interactions between CHRAC17 and γH2AX under the control of ATM signaling. **a** Green fluorescent protein (GFP)-chromatin assembly complex protein 17 (CHRAC17) accumulated at double-strand break (DSB) sites immediately after induction of DSB by laser micro-irradiation in H1299 cells. After laser micro-irradiation, cells were incubated for 10 min and immunostained with anti-γH2AX. **b** Results of chromatin immunoprecipitation (ChIP) analysis with immunoglobulin G (control) or antibodies against γH2AX, or CHRAC17 performed 0 or 12 h after transfection of the I-SceI expression plasmid into dA3-1 H1299 cells. The relative enrichment of proteins at each time point is presented, showing CHRAC17 recruitment at DSB sites. **c** Accumulation of GFP-CHRAC17 at DSB sites was not affected by knockdown of ATP-dependent chromatin assembly and remodeling factor 1 (ACF1). H1299 cells expressing GFP-CHRAC17 were laser micro-irradiated and subjected to real-time recording of protein accumulation at laser-irradiated sites. (left) Quantification of real-time recording of signal intensity on laser-micro-irradiated sites. (right) Intensity of GFP-CHRAC17 at 200 s after laser micro-irradiation. Data are presented as mean ± standard deviation of two independent biological replicates. **d** Knockdown of CHRAC17 reduced the accumulation of GFP-ACF1 at DSB sites. H1299 cells expressing GFP-ACF1 were subjected to laser micro-irradiation and real-time recording of protein accumulation at laser-micro-irradiated sites. (left) Quantification of real-time recording of signal intensity on laser-micro-irradiated sites by FLUOVIEW software (Olympus). (right) Intensity of GFP-ACF1 at 200 s after laser micro-irradiation. Data are presented as mean ± standard deviation of two independent biological replicates. **e** Knockdown efficiency of CHRAC17 is shown on a western blot. **f** Schematic diagrams of CHRAC17-deletion mutants. **g** The recruitment of CHRAC17 deletion mutants at DSB sites. H1299 cells expressing CHRAC17 deletion mutants were subjected to laser micro-irradiation and real-time recording of protein accumulation at laser-micro-irradiated sites. (Upper) Representative images. (bottom) Quantification of real-time recording of signal intensity on laser-micro-irradiated sites by FLUOVIEW software (Olympus). Intensity of GFP-CHRAC17 at 200 s after laser micro-irradiation. Data are presented as mean ± standard deviation of two independent biological replicates. **h** ATM signaling promotes CHRAC17 recruitment to DSB sites. H1299 cells expressing GFP-CHRAC17 were subjected to laser micro-irradiation and real-time recording of protein accumulation at laser-micro-irradiated sites. Quantification of real-time recording of signal intensity on laser-micro-irradiated sites by FLUOVIEW software (Olympus). Intensity of GFP-CHRAC17 at 60 s after laser micro-irradiation. All statistical analyses were performed using GraphPad Prism, with significance defined as *P* < 0.05. *P < 0.05, **P < 0.01, ***P < 0.001, ****P < 0.0001 (one-way analysis of variance). Scale bar: 10 μm. Abbreviations: Ai, inhibitor of ATM; ns, not significant.

In the context of cellular functions, including DSB repair, the functional relationship between the CHRAC15-17 and SNF2H-ACF1 catalytic subunit complex is poorly understood. It can be expected that CHRAC17 would accumulate at DSB sites because ACF1 binds the CHRAC15-17 complex via direct interaction with CHRAC17^10,11^. We previously demonstrated that SNF2H-ACF1 catalytic subunits accumulate at DSB sites via ACF1^5^. Similar to one of the catalytic subunits, GFP-ACF1 (Fig. 1a, lower) GFP-CHRAC17 (Fig. 1a, upper) accumulates at DSB sites immediately after induction of DSBs by laser micro-irradiation. On the other hand, GFP-CHRAC15 was weakly recruited to laser micro-irradiated DSB sites (Supplementary Fig. S1a).

Previously, we have shown that the cut efficiency of DSBs and γH2AX accumulation at DSB sites around 400 bp in dA3-1 H1299 cells increased at 12 h after transfection with the I-SceI expression plasmid (Fig. 1b, upper)^6,7^. Therefore, using dA3-1 H1299 cells, the ChIP assay was performed at 12 h post-I-SceI transfection-supported fluorescence studies, showing that CHRAC17 was recruited at DSB sites (Fig. 1b, left) with increased γH2AX formation (Fig. 1b, right).

Using published siRNAs targeting ACF1^5^, we observed the recruitment of GFP-CHRAC17 in ACF1 knockdown cells by live imaging analysis (Fig. 1c, left). We found that ACF1 knockdown did not reduce CHRAC17 accumulation at the DSB sites (Fig. 1c, right). Alternatively, live imaging of GFP-ACF1 at DSB sites in CHRAC17 knockdown cells (Fig. 1d, left) suggested that CHRAC17 knockdown reduced the accumulation of ACF1 (Fig. 1d, right and 1e). In addition, CHRAC17 knockdown reduced SNF2H accumulation at DSB sites (Supplementary Fig. S1b). CHRAC17 has been previously shown to bind to CHRAC15 and form an H2A/H2B-type histone-fold protein complex that promotes the chromatin-remodeling activity of ACF1-SNF2H^10,11^. Here, knockdown of CHRAC15 resulted in reduced recruitment of CHRAC17 at DSB sites (Supplementary Fig. S1c). These results suggest that CHRAC17 is recruited at DSB sites with CHRAC15, where it subsequently recruits ACF1-SNF2H, the catalytic subunit of CHRAC.

### CHRAC17 accumulation at DSB sites occurs via the H2B-type histone-fold domain, regulated by ataxia telangiectasia mutated (ATM) signaling

Next, we examined which domain of CHRAC17 is required for its recruitment to DSB sites. The structure of CHRAC17 contains H2B-type histone-fold domains in the N-terminal region (CHRAC17-N) and a high content of charged residues in the C-terminal region (CHRAC17-C), through which it binds to DNA and histone H3-H4^10,11,13^ (Fig. 1f). Deletion of the N- or C-terminal regions of CHRAC17 prevented its localization in the nuclei and therefore accumulation at DSB sites (Supplementary Fig. S1d), and therefore we fused NLS to GFP-CHRAC17 and its deletion mutants. Although GFP-Ve did not accumulate at DSB sites, GFP-CHRAC17-WT was recruited at DSB sites (Fig. 1g). Despite our observation of localization of GFP-CHRAC17-C to the nuclei, accumulation at DSB sites was not observed (Fig. 1g). In contrast, GFP- CHRAC17-N was equivalent to WT in terms of accumulation at DSB sites (Fig. 1g). Therefore, our findings suggest that the H2B-type histone-fold domain of CHRAC17—which lacks the DNA- and histone H3-H4-binding domain—is efficiently recruited at DSB sites.

It has been proposed that SWI/SNF, recruited to DSB sites via γ-H2AX, promotes further ATM-mediated γH2AX formation in surrounding chromatin by increasing nucleosome accessibility^14^. We found that knockdown of BRM, a catalytic SWI/SNF subunit, reduced CHRAC17 recruitment to DSB sites (Fig. S1e), suggesting that CHRAC17 recruitment depends on SWI/SNF-mediated chromatin remodeling and/or ATM signaling. Recruitment of GFP–CHRAC17 to DSB sites was also reduced by an ATM inhibitor (Fig. 1h), whereas inhibition of DNA-PKcs or ATR had no effect (Supplementary Fig. S1f). ATM inhibition reduced phosphorylation of ATM and its downstream target, CHK2, after IR-induced DSBs (Supplementary Fig. S2a). Similarly, ATM knockdown decreased ATM and CHK2 phosphorylation following IR (Supplementary Fig. S2b), supporting the inhibitor results.

Together, these findings suggest that SWI/SNF-mediated chromatin remodeling facilitates CHRAC17 recruitment but is insufficient without ATM signaling.

### The N-terminal region of CHRAC17 is sufficient for promoting the recruitment of BRCA1 and RAD51 to DSB sites

**Fig. 2:**
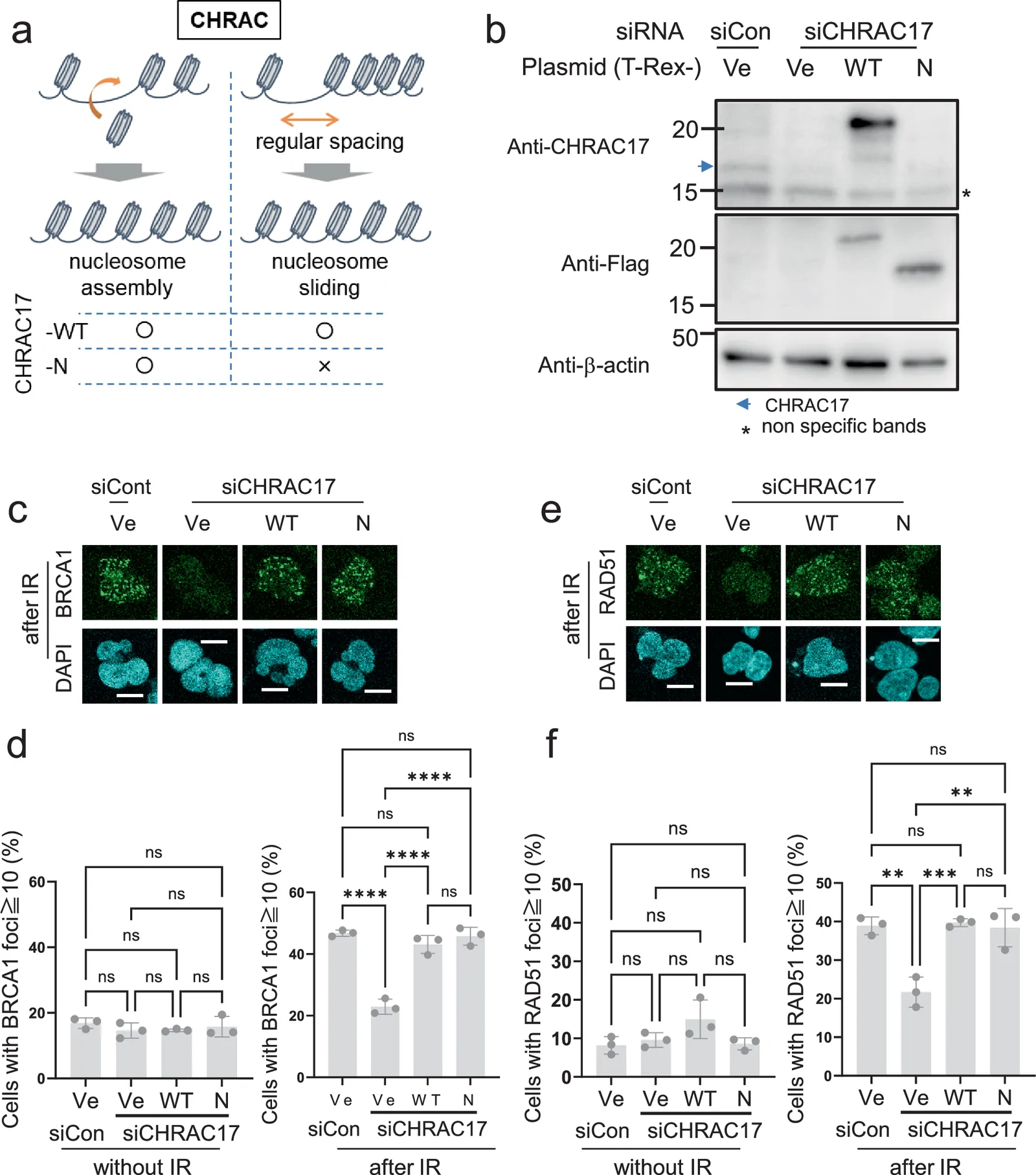
The CHRAC-N mutant promotes recruitment of BRCA1 and RAD51 at DSB sites. **a** ATP-dependent chromatin-remodeling activities of chromatin assembly complex (CHRAC)—nucleosome assembly and nucleosome sliding—have been detected in vitro. Wild-type chromatin assembly complex protein 17 (CHRAC17-WT) promotes both nucleosome assembly and sliding, but the CHRAC17-N mutant promotes nucleosome assembly only. **b** Western blot analysis of CHRAC17-WT and CHRAC17-N expression. HEK293 T-REx cells expressing FLAG-vector (Ve), FLAG-CHRAC17-WT (WT), or FLAG-CHRAC17-N (N) were transfected with siRNA targeting the untranslated region of CHRAC17 (siCHRAC17-UTR). Whole-cell extracts were analyzed by Western blot with the indicated antibodies. c,d CHRAC17-N promotes BRCA1 recruitment to DSB sites. HEK293 T-REx cells expressing vector (Ve), CHRAC17-WT (WT), or CHRAC17-N (N) were transfected with siCHRAC17-UTR and treated with or without 2 Gy IR. After 2 h, cells were fixed and immunostained with anti-BRCA1 antibody. **c** Representative images. **d** Percentage of cells with ≧10 BRCA1 foci. Data are presented as mean ± SD from three biological replicates. **e**,**f** CHRAC17-N promotes RAD51 recruitment to DSB sites. HEK293 T-REx cells expressing vector (Ve), CHRAC17-WT (WT), or CHRAC17-N (N) were transfected with siRNA targeting the untranslated region of the CHRAC17 transcript (siCHRAC17-UTR), and then were treated without and with 2 Gy IR. After 2 h, the cells were subsequently fixed and immunostained with anti-RAD51 antibodies. **e** Representative images. **f** Percentage of cells with ≧10 RAD51 foci. Data are presented as the mean ± standard deviation of three independent biological replicates. All statistical analyses were performed using GraphPad Prism, with significance defined as *P* < 0.05. *P < 0.05, **P < 0.01, ***P < 0.001, ****P < 0.0001 (one-way analysis of variance). Scale bar: 10 μm. Abbreviations: ns, not significant.

Two different SNF2H ATPase-dependent remodeling activities of CHRAC have been detected in vitro: nucleosome assembly and nucleosome sliding, and the only N-terminal histone-fold domain of H2B in CHRAC17 has been shown to promote nucleosome assembly but not nucleosome sliding (CHRAC17-N) (Fig. 2a)^1,10,11,15–17^. However, it was previously unclear for which in vivo cellular events require these two activities. We examined whether the N-terminal mutant of CHRAC17 interacts with SNF2H. Immunoprecipitation analyses showed that CHRAC17-N, like CHRAC17-WT, retained interaction with SNF2H (Supplementary Fig. S2c), consistent with reports that CHRAC17-N interacts with ACF1, a binding partner of SNF2H^10^.

We constructed Flip-in-TREx-CHRAC17-WT and Flip-in-TREx-CHRAC17-N cells, which stably expressed FLAG-tagged CHRAC17-WT and CHRAC17-N, respectively (Supplementary Fig. S3a). Knockdown of endogenous CHRAC17 using siRNA targeting the untranslated region of the CHRAC17 transcript (siCHRAC17-UTR), which has been used previously^13^, caused reduced endogenous expression of the CHRAC17 protein but did not reduce exogenous expression of CHRAC17-WT and CHRAC17-N (Supplementary Fig. S3a). While it has been reported that the knockdown of ACF1 reduced the recruitment of HR factors, BRCA1 and RAD51, at DSB sites, we found that knockdown of CHRAC17 (siCHRAC17 + Ve) reduced the recruitment of BRCA1 to DSB sites compared with control cells (siCont + Ve) following ionizing radiation (IR) treatment (Supplementary Fig. S3b and S3c). Although WT and N were overexpressed relative to endogenous CHRAC17 expression, their expression rescued the recruitment of BRCA1 at DSB sites to the same level as control cells (siCont+Ve) (Supplementary Fig. S3c). We also found that WT and N rescued the percentage of cells with BRCA1 foci ≧10. These analyses used traditional foci counting methods, which showed that WT and N rescued the recruitment of BRCA1 at DSB sites in siCHRAC17 knockdown cells (Supplementary Fig. S3d).

Next, we constructed TREx-CHRAC17 cells, in which the expression level of exogenous CHRAC17 protein is controlled via addition of doxycycline (Fig. 2b). We found that knockdown of CHRAC17 (siCHRAC17 + Ve) reduced the recruitment of BRCA1 to DSB sites compared with control cells (siCont + Ve) after IR treatment. Expression of WT (siCHRAC17 + WT) restored the recruitment of BRCA1 levels to that of control cells (Figs. 2c, d), as did CHRAC17-N expression (siCHRAC17 + N) in CHRAC17 knockdown cells (Figs. 2c, d). In addition, knockdown of CHRAC17 (siCHRAC17 + Ve) reduced the recruitment of RAD51 to DSB sites compared with control cells (siCont + Ve), and expression of WT and N (siCHRAC17 + WT and + N) restored the recruitment of RAD51 levels to that of control cells (Figs. 2e, f). Consistent with efficient BRCA1 and RAD51 recruitment, expression of WT and N (siCHRAC17 + WT and +N) restored DNA end resection to control levels (Supplementary Fig. S4a). Furthermore, expressions of WT and N (siCHRAC17 + WT and +N) restored recruitment of RAD54, a downstream factor of RAD51, to control levels (Supplementary Fig. S4b, c). These results suggest that the N-terminal region of CHRAC17 is sufficient to promote recruitment of BRCA1, RAD51, and the downstream factor RAD54 to DSB sites.

We also generated a T-REx–CHRAC17 C-terminal mutant plasmid designed for doxycycline-inducible expression. We constructed two independent plasmids, verified their sequences, and transfected them into T-REx 293 cells, but we were unable to detect expression of the CHRAC17 C-terminal mutant protein, although the CHRAC17-WT plasmid was readily expressed (Supplementary Fig. S4d). We next generated a Flp-In T-REx–CHRAC17 C-terminal mutant plasmid for stable expression under the CMV promoter. Again, we constructed two independent plasmids, verified their sequences, and transfected them into T-REx 293 cells, but we could not detect mutant protein expression, whereas CHRAC17-WT was readily expressed (Supplementary Fig. S4d). We also attempted to generate stable T-REx cell lines on three separate occasions, but still did not detect mutant protein expression (Supplementary Fig. S4e). Given its small size (~8 kDa), the CHRAC17 C-terminal mutant protein may be unstable, difficult to express, or toxic to cells. Although we could not assess its phenotype due to lack of expression, it failed to accumulate at DSB sites, suggesting it is unlikely to function in DSB repair.

### The N-terminal region of CHRAC17 does not facilitate RAD51-mediated recombination processes

**Fig. 3:**
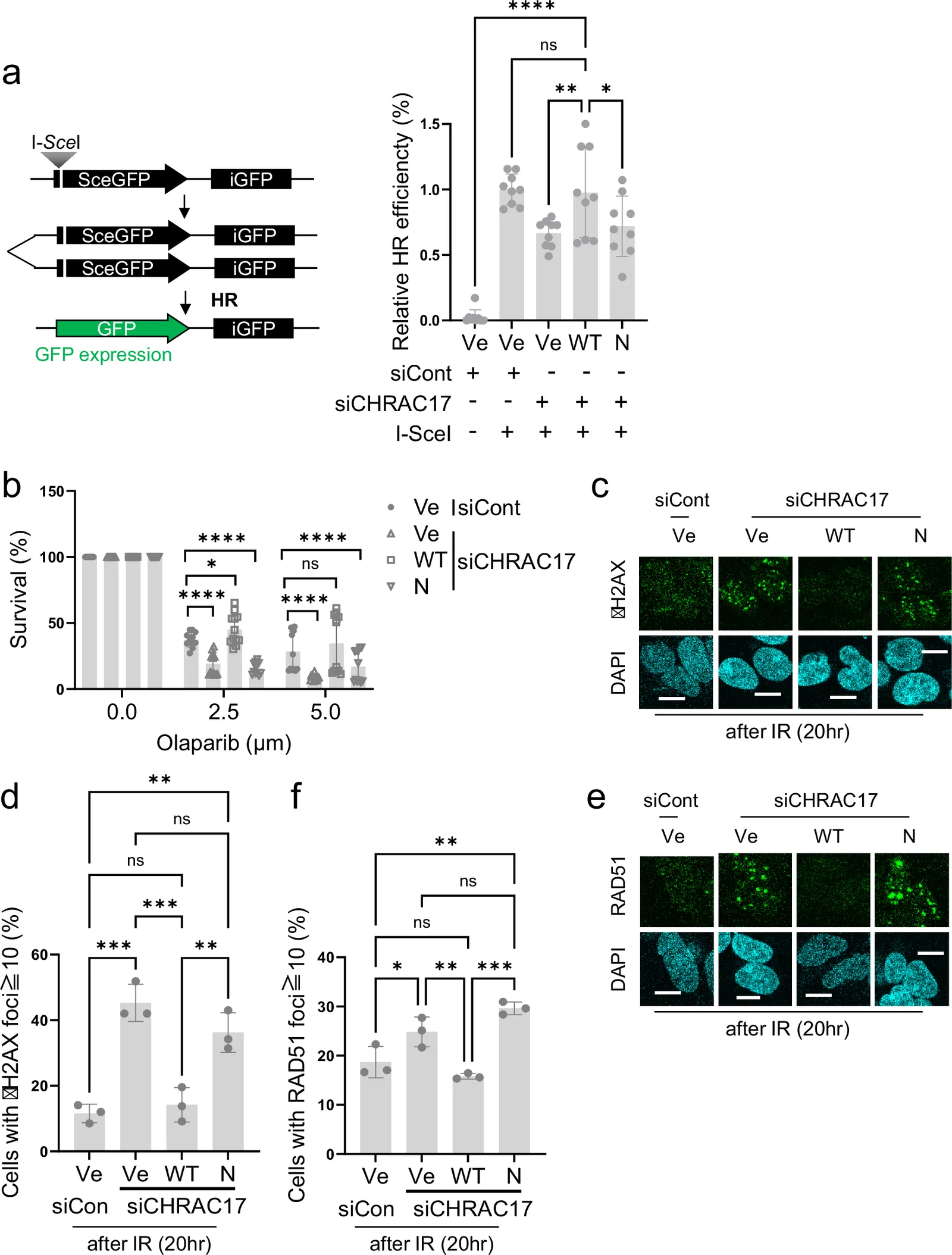
The CHRAC-N mutant failed to promote RAD51-mediated recombination processes and the release of RAD51 from DSB. **a** Schematic diagrams of the DR-GFP HR assay report system (left). The N-terminal domain of CHRAC17 (CHRAC17-N) shows reduced HR efficiency compared with wild-type (CHRAC17-WT), as indicated by the GFP-positive cell fraction in HeLa cells (right). Data are presented as mean ± standard deviation of three independent biological replicates. **b** CHRAC17-N increased the sensitivity to olaparib compared with CHRAC17-WT (according to analysis with PrestoBlue^TM^). Data are presented as mean values ±standard deviation from two independent replicates. Data are presented as mean ± standard deviation of three independent biological replicates. **c**,**d** Expression of CHRAC17-N in CHRAC17 knockdown cells (CHRAC17-N) exhibited insufficient DSB repair compared with expression of CHRAC17-WT in CHRAC17 knockdown cells (CHRAC17-WT), and control cells (siCont + Ve). HEK293 T-REx cells expressing vector (Ve), CHRAC17-WT (WT), or CHRAC17-N (N) were transfected with siRNA targeting the untranslated region of the CHRAC17 transcript (siCHRAC17-UTR) and treated with 2 Gy IR. Twenty hours after IR treatment, cells were fixed and immunostained with anti-γH2AX antibody. **c** Representative images. **d** Percentage of cells with ≧10 γH2AX foci. Data are presented as mean ± standard deviation from three independent biological replicates. **e**,**f** HEK293 T-REx cells expressing vector (Ve), CHRAC17-WT (WT), or CHRAC17-N (N) were transfected with siRNA targeting the untranslated region of the CHRAC17 transcript (siCHRAC17-UTR) and then treated with 2 Gy IR. Twenty hours after IR treatment, cells were fixed and immunostained with anti-RAD51 antibody. **e** Representative images. **f** Percentage of cells with ≧10 RAD51 foci. Data are presented as mean ± standard deviation of three independent biological replicates. All statistical analyses were performed using GraphPad Prism, with significance defined as *P* < 0.05. *P < 0.05, **P < 0.01, ***P < 0.001, ****P < 0.0001 (one-way analysis of variance). Scale bar: 10 μm. Abbreviations: ns, not significant.

We next examined whether the N-terminal region of CHRAC17, which promotes the nucleosome-assembly activity of CHRAC, alone is sufficient for complete HR. Knockdown of CHRAC17 (siCHRAC17 + Ve) reduced HR activity (DR-GFP assay) compared with control cells (siCont + Ve)^18^ (Fig. 3a). Expression of CHRAC17-WT restored this activity; however, expression of CHRAC17-N did not restore HR activity in CHRAC17-knockdown cells. Furthermore, knockdown of CHRAC17 (siCHRAC17 + Ve) increased the sensitivity to poly-(adenosine diphosphate-ribose) polymerase inhibitor (PARPi) compared with control cells (siCont + Ve). Expression of CHRAC17-WT restored resistance of CHRAC17 knockdown cells, while expression of CHRAC17-N did not restore it completely (Fig. 3b). Thus, although the N-terminal region of CHRAC17 promotes the recruitment of BRCA1 and RAD51 to DSB sites, it alone is insufficient for complete HR. Consistent with this finding, γH2AX foci were absent in control cells (siCont + Ve) and CHRAC17-WT-expressing CHRAC17 knockdown cells (siCHRAC17 + WT) during a 20-h incubation after IR treatment, but they were detected in CHRAC17-knockdown (siCHRAC17 + Ve) cells and in cells expressing CHRAC17-N (siCHRAC17 + N) (Figs. 3c, d). Thus, the N-terminal region of CHRAC17 alone was insufficient to promote DSB repair.

Consistent with this finding, RAD51 foci were absent in control cells (siCont + Ve) and CHRAC17-WT-expressing CHRAC17 knockdown cells (siCHRAC17 + WT) during a 20-h incubation after IR treatment, but were detected in CHRAC17-knockdown (siCHRAC17 + Ve) cells and in cells expressing CHRAC17-N (siCHRAC17 + N) (Figs. 3e, f), suggesting that RAD51-mediated recombination processes are disturbed in the CHRAC17-N cells. This indicates that the N-terminal region of CHRAC17 fails to facilitate RAD51-mediated recombination processes for complete DSB repair.

A site-specific homologous recombination assay (Supplementary Fig. S5a) was performed where a defined DSB was induced by CRISPR/Cas9, and successful BRCA1- and RAD51-mediated HR was quantified by real-time PCR using locus-specific primers^19^. This assay directly measures the completion of the HR process at the level of DNA repair product formation. As shown in Supplementary Fig. S5b, cells expressing CHRAC17-N had a significant HR completion defect compared with cells expressing CHRAC17-WT.

### Polε complex promotes HR

**Fig. 4:**
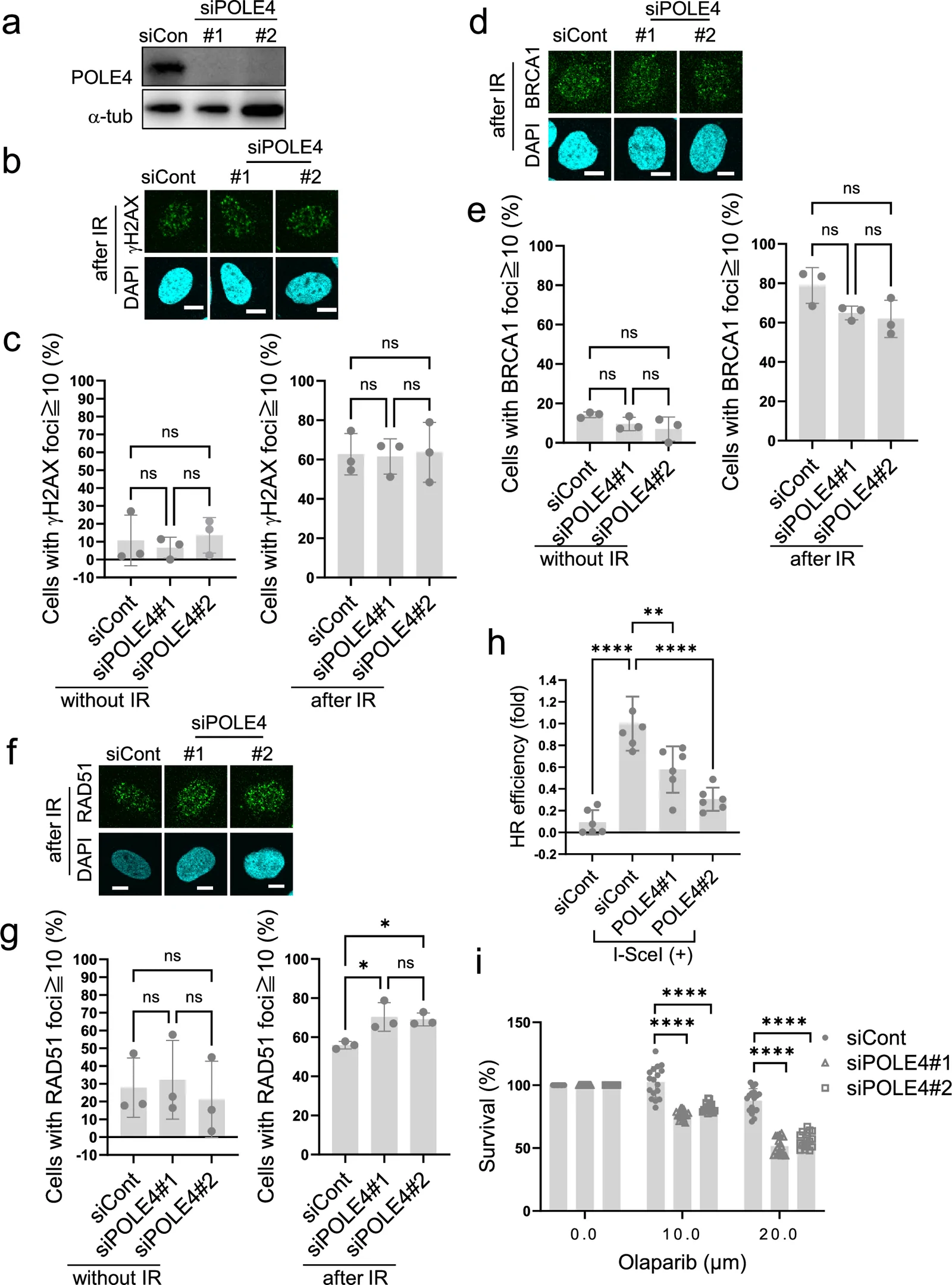
Polε complex is not required for the recruitment of γH2AX foci, BRCA1 and RAD51 at DSB site, but is required for RAD51-mediated recombination processes. **a** Western blot analysis of POLE4 knockdown. **b**,**c** POLE4 knockdown did not affect γH2AX recruitment to DSB sites. U2OS cells transfected with siRNA were treated with or without 2 Gy IR. After 30 min, cells were fixed and immunostained with anti-γH2AX antibody. **b** Representative images. **c** Percentage of cells with≧10 γH2AX foci. Data are presented as mean ± standard deviation from three independent biological replicates. **d**,**e** POLE4 knockdown did not affect BRCA1 recruitment to DSB sites. Cells were treated as above and immunostained with anti-BRCA1 antibody after 2 h. **d** Representative images. **e** Percentage of cells with ≧10 BRCA1 foci. Data are presented as mean ± standard deviation from three biological replicates. **f**,**g** POLE4 knockdown did not affect RAD51 recruitment to DSB sites. Cells were treated as above and immunostained with anti-RAD51 antibody after 2 h. **f** Representative images. **g** Percentage of cells with ≧10 RAD51 foci. Data are presented as mean ± standard deviation from three biological replicates. **h** POLE4 knockdown reduces HR efficiency compared with control cells. HR efficiency was measured as the GFP-positive fraction using a DR-GFP reporter assay in HeLa cells. Data are presented as mean ± standard deviation from three biological replicates. **i** POLE4 knockdown increased sensitivity to olaparib compared with control cells, measured using PrestoBlue™. Data are presented as mean ± standard deviation from three biological replicates. All statistical analyses were performed using GraphPad Prism, with significance defined as *P* < 0.05 (*P < 0.05, **P < 0.01, ***P < 0.001, ****P < 0.0001; one-way ANOVA). Scale bar: 10 μm. Abbreviations: ns, not significant.

CHRAC17, another name for POLE3, together with POLE4, binds to the catalytic subunit of the Pol ε complex (composed of POLE1 and POLE2) and functions as a histone chaperone within Pol ε^13^. Although the catalytic subunit is essential for DNA replication and cell growth, knockdown of POLE4 or CHRAC17 /POLE3 does not affect DNA replication or cell growth^13^^,^ which has also been reported for the yeast homologs, DPB3 and DPB4^20^. Because CHRAC17 is involved in both CHRAC and the Pol ε complex, knockdown causes loss of function of both functions. Therefore, we use siRNA targeting POLE4, to examine the function of Pol ε at DSB sites. We found that POLE4 knockdown did not affect the formation of γH2AX foci (Fig. 4a–c) or BRCA1 recruitment at DSB sites after IR treatment (Fig. 4d, e). Interestingly, POLE4 knockdown did not affect the recruitment of RAD51 at DSB sites after IR treatment but increased its recruitment more than that observed in control cells (Fig. 4f, g). This suggests that the Pol εcomplex does not influence recruitment, rather than stalling, of RAD51 at DSB sites, implying that RAD51-mediated recombination processes are disturbed. In addition, POLE4 (siPOLE4#1 and siPOLE4#2) knockdown reduced HR activity (Fig. 4h), and the re-expression of POLE4 in cells treated with POLE4#2, which targets the untranslated region of the POLE4 transcript, restored this activity (Supplementary Fig. S6a). In addition, POLE4 knockdown increased the sensitivity to PARPi (Fig. 4i), further suggesting that the Polε complex is required for HR processes.

**Fig. 5:**
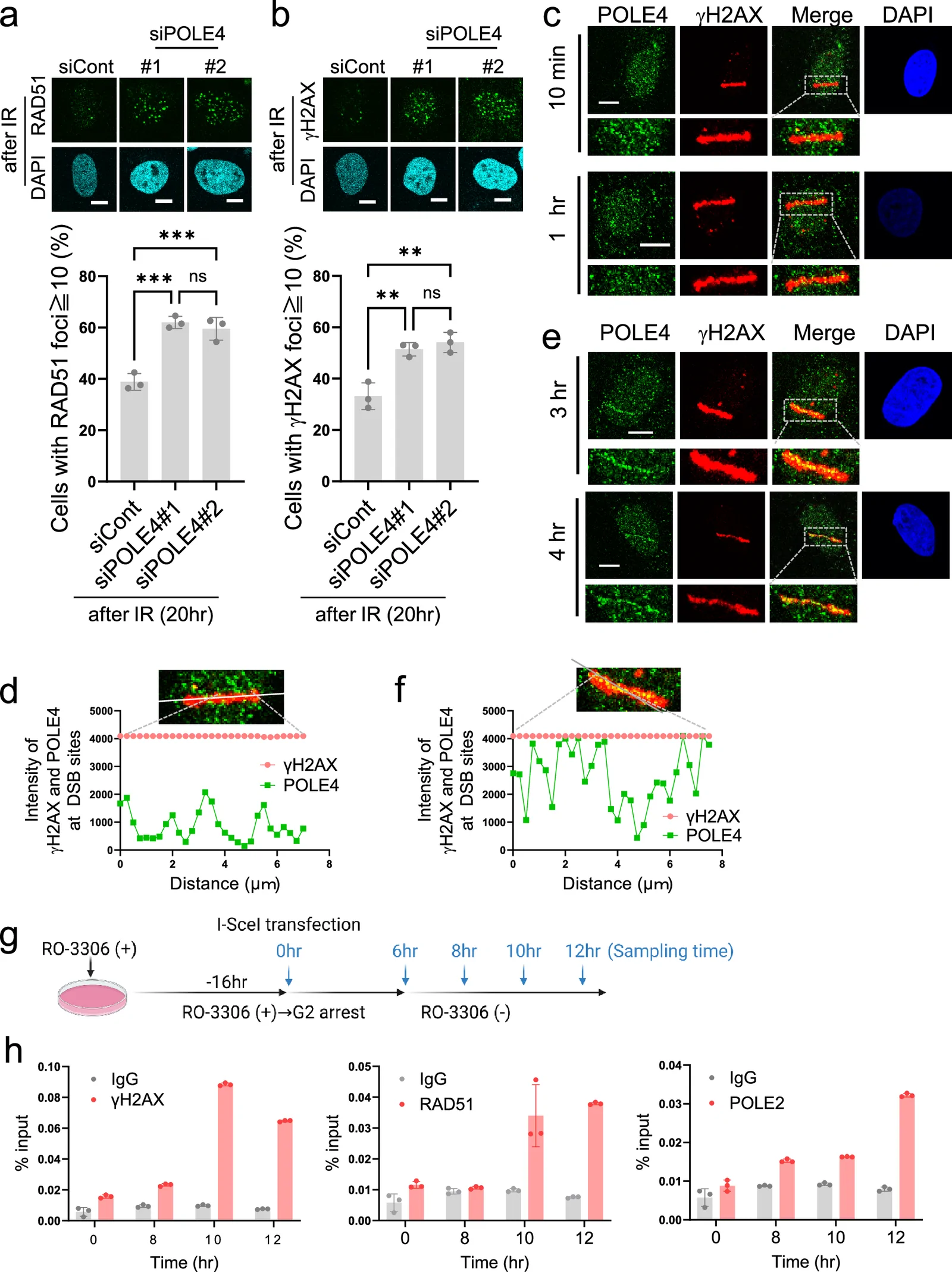
Pol ε is recruited to double-strand break sites. **a** POLE4 knockdown retains RAD51 foci after 20 h incubation following IR treatment. U2OS cells were transfected with each siRNA and then treated with 2 Gy IR. After 20 h, cells were fixed and immunostained with anti- RAD51 antibody. (upper) Representative images. (lower) Percentage of cells with ≧10 RAD51 foci. Data are presented as mean ± standard deviation from three independent biological replicates. **b** POLE4 knockdown retains γH2AX foci after 20 h incubation following IR treatment. Cells were treated as above and immunostained with anti-γH2AX antibody. (upper) Representative images. (lower) Percentage of cells with ≧10 γH2AX foci. Data are presented as mean ± standard deviation from three independent biological replicates. **c**,**d** **c** Representative images showing that POLE4 is not recruited to DSB sites at 10 min and 1 h after irradiation. **d** Quantification of signal intensity around laser micro-irradiated sites at 10 min plotted against distance using ImageJ. **e**,**f** **e** Representative images showing that POLE4 is not recruited to DSB sites at 3 h and 4 h after irradiation. **f** Quantification of signal intensity around laser micro-irradiated sites at 3 h plotted against distance using ImageJ. **g** Experimental workflow of ChIP assay. **h** Chromatin immunoprecipitation (ChIP) analysis was performed using immunoglobulin G (control) or antibodies against γH2AX, RAD51, or POLE2 at 0, 8, 10, and 12 h after transfection of the I-SceI expression plasmid into G2-phase DR-GFP HeLa cells. Relative enrichment at each time point is shown. All statistical analyses were performed using GraphPad Prism, with significance defined as *P* < 0.05. *P < 0.05, **P < 0.01, ***P < 0.001, ****P < 0.0001 (one-way analysis of variance). Scale bar: 10 μm. Abbreviations: ns, not significant.

On the other hand, the decrease in RAD51 and γH2AX foci observed in control cells after 20-h IR irradiation was not observed in POLE4-knockdown cells (Figs. 5a, b), suggesting that RAD51-mediated HR processes are disturbed and DSBs remain unrepaired. A site-specific homologous recombination assay in POLE4 knockdown cells was performed (Supplementary Fig. S6b). As shown in Fig. S6b, POLE4-depleted cells exhibited a defect in BRCA1- and RAD51-mediated HR completion compared with control cells.

### Polε complex is recruited at DSB sites

Although CHRAC17 was rapidly recruited to DSB sites (Fig. 1a), POLE4 was not recruited within 1 h after laser-induced DSBs (Fig. 5c), and its intensity did not increase at DSB sites (Fig. 5d). However, a weak POLE4 signal was detected 3–4 h after DSB induction (Fig. 5e), accompanied by increased POLE4 intensity at DSB sites (Fig. 5f). Knockdown of CHRAC17 reduced POLE4 recruitment to DSB sites (Supplementary Fig. S7a, b). These results suggest that Polε complex recruitment to DSB sites occurs during the late steps of HR and is regulated by CHRAC17.

We next performed a ChIP assay to detect the recruitment of POLE2 at DSB sites in DR-GFP HeLa cells, which are synchronized in the G2 phase (Supplementary Fig. S7c) to eliminate the possibility of detecting POLE2 recruitment in DNA replication forks (Fig. 5g). The ChIP assay revealed that DSBs (detected by γH2AX accumulation) occur gradually during the 8-h incubation after I-SceI transfection. We washed out RO3306 for 6 h after I-SceI transfection, prior to the induction of DSBs in order to eliminate any possibility of the Cdk1 inhibitor affecting HR processes. γH2AX recruitment was found to increase at 10 h after I-SceI transfection (Fig. 5h, left), and RAD51 was also recruited at this time point (Fig. 5h, middle). We also found that POLE2 was recruited at DSB sites at 10–12 h after I-SceI transfection (Fig. 5h, right). Furthermore, we performed a ChIP time-course analysis to examine the kinetics of CHRAC17 recruitment to DSB sites following DNA damage (Supplementary Fig. S7d). We examined whether POLE2 recruitment depends on the homologous recombination factor RAD51. As shown in Supplementary Fig. S7e, RAD51 knockdown reduced POLE2 recruitment, suggesting that POLE2 functions downstream of RAD51.

We next examined whether CHRAC17-N promotes Polε recruitment to DSB sites. As shown in Supplementary Fig. S7f, CHRAC17-WT efficiently supported Polε accumulation at DSBs, whereas CHRAC17-N did not. These results indicate that CHRAC17-dependent nucleosome remodeling is required for Polε recruitment during HR repair.

## Discussion

### CHRAC17, also known as POLE3, plays important roles in DSB repair in both CHRAC in the ISWI chromatin remodeler, and the Pol ε complex

The present study demonstrates that the chromatin remodeler CHRAC and DNA polymerase, Polε, are crucial for HR, and CHRAC17 has important roles within these complexes. CHRAC17 first facilitates the nucleosome-assembly activity of CHRAC to recruit HR factors to DSB sites, and subsequently promotes additional chromatin-remodeling activities, including the nucleosome-sliding activity previously demonstrated by in vitro studies^1,9–11^, thereby enabling efficient HR. Then, Polε complex is recruited at DSB sites through CHRAC17, promoting RAD51-mediated recombination processes.

### CHRAC17 facilitates the sequential nucleosome-assembly and additional nucleosome-remodeling activity, including nucleosome sliding, of CHRAC to achieve efficient HR

In CHRAC, CHRAC17 is recruited at DSB sites via its H2B histone-fold motif, and CHRAC17 plays an important role in recruiting ACF1 and SNF2H, the catalytic subunit complexes of the ISWI family, to DSB sites (Fig. 1d and Supplementary Fig. S1b). We have previously reported that ACF1 accumulates at DSB sites via its WAC (WSTF/Acf1/cbp146) domain, where it subsequently recruits SNF2H to DSB sites^5^, while the WAC domain binds CHRAC17 directly^10,11^. Combined with knowledge from previous studies, our findings indicate that the SNF2H-ACF1 catalytic subunit complex accumulates at DSBs via the H2A-H2B-type histone-fold protein complex, CHRAC15-17.

The recruitment of CHRAC17 to DSB sites requires the SWI/SNF catalytic subunit BRM (Fig. S1e), suggesting that SWI/SNF chromatin remodeling activity plays an important role in CHRAC17 recruitment at DSB sites. The SWI/SNF catalytic subunits BRG1 and BRM have been shown to interact with nucleosomes containing γH2AX and acetylated H3^14^, and previous studies have proposed that recruited SWI/SNF facilitates ATM-mediated H2AX S139 phosphorylation and DNA repair, likely by increasing nucleosome accessibility around DSBs^14,21^. In addition, we found that ATM inhibition reduced CHRAC17 recruitment to DSB sites (Fig. 1h and Supplementary Fig. S1f). Together, these results suggest that SWI/SNF-mediated chromatin remodeling alone is insufficient for CHRAC17 recruitment, and that ATM signaling is additionally required for efficient recruitment of CHRAC17 to DSB sites.

Previously, two different SNF2H ATPase-dependent remodeling activities related to CHRAC have been detected via in vitro studies: nucleosome assembly and nucleosome sliding. However, although the N-terminal histone-fold domain of H2B in CHRAC17 is the only one that has been shown to promote nucleosome-assembly activity, it does not promote nucleosome-sliding activity^1,10,11,15–17^. We show here that only N-terminal histone-fold domain of H2B in CHRAC17 promotes DNA end resection (Supplementary Fig. S4a) and recruitment of HR factors, such as BRCA1, RAD51(Fig. 2), and RAD54 (Supplementary Fig. S4b, c), at DSB sites, but does not promote RAD51-mediated recombination processes to complete DSB repair (Fig.3, Supplementary Fig. S5b). Based on previous in vitro studies^1,9–11^, our results imply that CHRAC17 promotes two chromatin-remodeling activities, nucleosome assembly and additional nucleosome-remodeling activity, including nucleosome sliding at different steps of the HR pathway. First, nucleosome assembly activity driven by CHRAC promotes recruitment of BRCA1 and RAD51 at DSB sites, and next, additional nucleosome-remodeling activity, including nucleosome sliding, driven by CHRAC facilitates RAD51-mediated recombination processes for the completion of HR. In vitro studies have shown that nucleosome assembly driven by ISWI could establish compacted chromatin, while ACF1 directly binds HP1 and loads it onto H3K9-methylated nucleosomes^22^. Recent studies have suggested that heterochromatin protein 1 (HP1)-mediated chromatin compacting is established at DSB sites to facilitate BRCA1-mediated HR and limit excessive DNA-end resection for efficient HR^12,23–28^. Although chromatin-remodeling complexes might be involved in establishing HP1-mediated chromatin compaction at DSB sites, the mechanism has thus far been unclear. Our results imply that CHRAC drives nucleosome assembly, thereby promoting chromatin compaction for efficient HR. Interestingly, another DSB remodeler—the SWI/SNF complex—increases DNA accessibility around DSB sites through nucleosome ejection or sliding, which preferentially relaxes chromatin enabling accumulation of γH2AX through ATM activation^21,29^. In contrast, the catalytic subunit of CHRAC, does not influence ATM activation or γH2AX accumulation at DSB sites, suggesting that ISWI does not affect early DSB responses^30–32^. Our data support previous studies, suggesting that SWI/SNF relaxes chromatin structure around DSBs to activate ATM signaling during the early DSB response, and after that, if HR is required for the repair of the DSB sites, CHRAC assembles nucleosomes to establish compacted chromatin, leading to BRCA1 recruitment, limiting DNA-end resection and efficient HR.

The present study also demonstrates that nucleosome assembly alone is insufficient for completion of the HR pathway, and that additional nucleosome-remodeling activity, including nucleosome sliding, is required to promote RAD51-mediated recombination and completion of HR (Figs. 2, 3). After the recruitment of RAD51 to DSB sites, RAD51 promotes the formation of synaptic complexes and D-loops for recombination^33^. Therefore, CHRAC-mediated nucleosome-remodeling activity may be required for these RAD51-mediated recombination processes after RAD51 binding at DSB sites, with subsequent release of RAD51 from DSB sites to complete HR. Interestingly, the T114Qfs*32 mutation—which resembles our CHRAC17-N mutant—has been found in stomach and breast cancer in the cBioPortal database (cBioPortal for Cancer Genomics). Thus, cancer cells harboring this mutation might be sensitive to PARPi because of defective HR.

### Roles of Polε complex in DSB repair

The question of whether Polε is involved in DSB repair and HR in humans remains unanswered^12^. The function of Polε is best characterized in *Saccharomyces cerevisiae*. Although yeast CHRAC17 (Dpb4) and POLE4 (Dpb3) are nonessential subunits and do not affect the processivity of Polε, *Dpb4* deletion mutants exhibit sensitivity to DSB-inducing agents^34^, and the catalytic subunits of yeast Polε are suggested to be involved in HR^12,35^.

POLE4 knockdown did not affect BRCA1 or RAD51 recruitment to DSB sites (Fig. 4e, h), suggesting that Polε is dispensable for early HR factor recruitment. CHRAC17 knockdown reduced POLE4 and POLE2 recruitment (Supplementary Fig. S7a, b, f), suggesting that the Polε complex is recruited via CHRAC17. In addition, POLE2 was recruited to DSB sites (Fig. 5h), and this recruitment was reduced by RAD51 knockdown (Supplementary Fig. S7e), suggesting that POLE2 functions downstream of RAD51. Furthermore, in a site-specific DSB repair assay, knockdown of POLE4 and CHRAC17 impaired completion of BRCA1- and RAD51-mediated HR (Supplementary Figs. S5b, 6b), indicating that both proteins are required for the late steps of HR and HR completion. After RAD51 stimulates synaptic complex and D-loop formation, HR is completed by DNA synthesis^36,37^. The DNA synthesis of yeast and human DNA polymerase δ (Pol δ) is well studied, with the latter shown to promote first-end synthesis (D-loop extension) with RAD51^12^. Because Polε is incapable of RAD51-mediated D-loop extension in vitro, Polε is hypothesized to be involved in second-end synthesis in HR^12^. Combined with the previous report that the histone chaperone CHRAC17-POLE4 promotes the DNA-synthesis-coupled nucleosome-assembly activity of the Polε complex^13^, our finding supports the postulation that CHRAC17-POLE4 promotes Polε-mediated DNA-synthesis-coupled nucleosome assembly in the HR pathway. Therefore, after RAD51/Polδ-mediated first-end synthesis, Polε is probably recruited by CHRAC17 to DSBs, and then Polε may promote second-end synthesis, with DNA synthesis-coupled nucleosome-assembly activity, and the release of RAD51 to promote HR. The polymerase activity of Polε is essential for cell viability, and so we were unable to investigate this function. However, mutation of the yeast CHRAC17/DPB4 exhibits severe repair defects, in contrast to a polymerase activity-deficient mutation of polε (*pol2-16*), suggesting that the nucleosome-assembly activity of CHRAC17-POLE4 may be more important in HR than Polε polymerase activity^12^. This could be attributable to the overlapping functions of Polε and Polδ in DNA synthesis during HR, while the histone chaperone activity of CHRAC17-POLE4 is essential and cannot be compensated to reconstitute nucleosomes on nascent naked DNA synthesized by DNA polymerase during HR

## Methods

### Materials availability

All materials generated in this study are available from the lead contact upon request.

### Establishment and culture of cell lines

We cultured H1299 and dA3-1 H1299 human lung cancer cells in RPMI1640 supplemented with 10% fetal bovine serum (FBS) at 37 °C with 5% CO_2_^7,38,39^_._ We cultured HeLa DR-GFP cells and U2OS cells (ATCC, Manassas, Virginia, USA, HTB-96) in DMEM supplemented with 10% FBS at 37 °C in 5% CO_2_^5^. We cultured HeLa cells in DMEM supplemented with 10% fetal bovine serum (FBS) at 37 °C with 5% CO_2._

Flip-In^TM^ T-REx^TM^ 293 cells and T-REx^TM^ 293 cells expressing FLAG-tagged CHRAC17 were established using the Flip-In^TM^ T-REx^TM^ system (Invitrogen), according to the manufacturer’s protocol, then cultured in DMEM supplemented with 10% FBS at 37 °C with 5% CO_2_^40^. We established cell lines that stably express CHRAC17-WT or CHRAC17-N harboring the FLAG-tag and NLS.

### Plasmid and siRNA transfection

We created two deletion mutant proteins in pEGFP-C1: One containing only the H2B-type histone-fold domains and the other only the C-terminal region (Fig. 1G). Nuclear location signal (NLS)-fused constructs of the vector (Ve), CHRAC17-WT, and deletion mutants were created to compare recruitment. Transient transfections were performed using Lipofectamine 2000 and Lipofectamine RNAiMAX (Thermo Fisher Scientific) according to the manufacturer’s protocol.

### siRNAs

We used a mixture of the following siRNAs: siPOLE3-UTR (Invitrogen ON-TARGET plus; Cat#J-008460-10-0005), siPOLE4 (Invitrogen Silencer Select Pre-Designed siRNA; Cat# s32215 and Cat# s32216). To target ATP-dependent chromatin assembly and remodeling factor 1 (ACF1), we used siRNA sequences that have been previously published^5–7,39^.

### Laser micro-irradiation

Laser micro-irradiation was performed using the FV-500 confocal scanning laser microscopy system (Olympus; Tokyo, Japan), as reported previously^5–8,39,41^ .

### Immunofluorescence staining and analysis of microscope-based imaging

Immunofluorescence staining protocol is described previously, with slight modifications^40,41^. siRNA-transfected cells grown on glass-bottom dishes were treated with 2 Gy IR or laser micro-irradiation. After incubation for the indicated time, cells were fixed by incubating with 3% formaldehyde/2% sucrose in phosphate-buffered saline (PBS) for 15 min at room temperature (RT) and permeabilized with 0.5% Triton X-100 for 5 min on ice. Cells were incubated with primary antibodies at RT, followed by incubation with secondary antibodies at RT. Counterstaining of DNA was carried out with 0.1 mg/ml of 4′, 6-diamidino-2-phenylindole (DAPI). Fluorescence images were acquired using an Olympus confocal microscope, FV3000 (Olympus). The recruitment of proteins at DSB sites was measured using FLUOVIEW software (Olympus). Graphs were constructed using GraphPad Prism version 10.4.2. Statistical analyses used to score IR-induced foci were performed using GraphPad Prism version 10.4.2. Antibodies used in the immunofluorescence staining were γH2AX (05-636; MilliporeSigma), BRCA1 (sc-6954, Santa Cruz Biotechnology), RAD51 (14961-1-AP, proteintec), RAD54 (ab11055, abcom), POLE3/CHRAC17 (A301-245A, Thermo Fisher Scientific), POLE4 (PA5-97283, Thermo Fisher Scientific).

### Survival assays

Cells were pretreated with siRNA and/or transformed with plasmid for 48 h prior to exposure to PARPis. Cell viability was measured using fluorescence by plating cells onto 96-well plates and analyzing surviving colonies using the PrestoBlue™ Cell Viability Reagent (Thermo Fisher Scientific).

### Western blot analysis

Harvested cells were washed with PBS and then lysed in lysis buffer (50 mM Tris-HCl (pH 7.5), 1 mM ethylenediaminetetraacetic acid, 150 mM NaCl, 1% NP-40, 0.1% sodium dodecyl sulfate (SDS), and 0.1% Na deoxycholate) containing 1 mM phenylmethylsulphonyl fluoride and phosphatase inhibitor cocktail (Roche Diagnostics; Indianapolis, IN, USA). After 30 min incubation on ice, samples were centrifuged for 10 min at 14,000 rpm at 4 °C. Supernatants were collected, then mixed with SDS sample buffer, boiled, and subjected to SDS-polyacrylamide gel electrophoresis followed by blotting onto a polyvinylidene difluoride membrane. The membrane was blocked overnight with Tris-buffered saline (TBS) containing 0.1% Tween 20 and 0.5% bovine serum albumin, then probed with primary antibodies. After washing with TBS containing 0.1% Tween 20, membranes were incubated with horseradish peroxidase-conjugated anti-mouse or anti-rabbit antibodies and treated with an enhanced chemiluminescence reagent (GE Healthcare; Tokyo, Japan). Antibodies used in the Western blot analysis were FLAG (F3165; Sigma-Aldrich, 1:3000), b-actin (A5441, Sigma-Aldrich, 1:3000), BRCA1 (sc-6954, Santa Cruz Biotechnology, 1:1000), POLE3/CHRAC17 (A301-245A, Thermo Fisher Scientific, 1:1000), POLE4 (PA5-97283, Thermo Fisher Scientific, 1:1000), CHRAC15 (A304-987A, Thermo Fisher, 1:1000), CHEK2 (13954-1-AP, proteintec, 1:1000), pCHEK2 (#2661, pCHEK2, 1:1000), ATM (27156-1-AP, proteintec, 1:1000), pATM (#5883, Cell signaling, 1:1000)

### HR assay

The HR assays that were performed have been previously published^5^. Briefly, DR-GFP HeLa cells (pre-transfected with siRNA for 48 h using Lipofectamine RNAiMAX) were transfected with the pCBASceI (RRID:Addgene 2647) plasmid (i.e., the I-SceI expression plasmid) using the Lipofectamine 2000 reagent. For fluorescence-activated cell sorting, cells were harvested by trypsinization, washed with PBS, then applied to the CytoFLEX (Beckman Colter). Enhanced-GFP-positive cells were counted using the built-in software.

### Chromatin immunoprecipitation

We synchronized DR-GFP HeLa cells in the G2 phase using a Cdk1 inhibitor (RO3306) for 16 h. We then transfected DR-GFP HeLa cells with the pCBASce (RRID: Addgene 2647) plasmid using Lipofectamine RNAMAX (Invitrogen). At the indicated time points after transfection, cells were incubated with 1% formaldehyde for 10 min at RT to cross-link proteins to DNA. The reaction was stopped by the addition of glycine. Cells were collected using a cell scraper and washed in ice-cold PBS. Chromatin Immunoprecipitation (ChIP) assays were performed using the ChIP-IT^®^ Express Enzymatic kit (Active Motif) according to the manufacturer’s protocol and previous reports^5–7,39^. Briefly, after DNA shearing, a portion of each sample was set aside for input control, and the remaining portions were subjected to immunoprecipitation overnight at 4 °C. Cross-links were reversed by incubating in a reverse cross-linking buffer for 15 min at 95 °C, then samples were de-proteinized for 1 h at 37 °C using proteinase K. Aliquots were subjected to quantitative polymerase chain reaction (PCR) using a real-time PCR system and GoTaq qPCR Master Mix (Promega). Precipitated DNA was analyzed using the following primers 400–500 bp upstream of the I-SceI site: 5′-CGTGCTGGTTATTGTGCTGTCTCATC-3′ (forward) and 5′-GTGCTGCATGCTTCTTCGGCA-3′ (reverse). The proportion of DNA from the test loci that cross-linked to proteins was calculated for each time point based on the quantity of DNA in the input control samples. Relative enrichment of a protein at the test sites was expressed as the amount of immunoprecipitated DNA divided by the amount of input DNA, which was then plotted as ratios against the values for 0 h. Antibodies used in the ChIP assay were γH2AX (05-636; MilliporeSigma) (RRID:AB_309864), POLE3/CHRAC17 (A301-245A, Thermo Fisher Scientific), and POLE2 (NBP1-87970; Novus) (RRID:AB_11060856).

### A site-specific homologous recombination assay

A site-specific homologous recombination assay was performed as previously described^19^. Briefly, cells were pretransfected with siRNA for 48 h using Lipofectamine RNAiMAX (Invitrogen) and harvested for qPCR 72 h after transfection with ACTB-Cas9 and donor vectors. Genomic DNA was extracted 72 h post-transfection, and qPCR was used to determine fusion gene abundance. Primer sequences are as follows: ACTB-ref-R3 (5′- GACTTAGTTGCGTTACACCCTTTCTTG -3′) and ACTB-common-R2 (5′- TGCAATCAAAGTCCTCGGCCAC -3′) for the reference allele of *ACTB*; GFP-F1 (5′-GTCCTGCTGGAGTTCGTGACCG-3′) and ACTB-common-R2 (5′- TGCAATCAAAGTCCTCGGCCAC -3′) for the fusion allele of *ACTB*.

### Immunoprecipitation

Immunoprecipitation was performed as previously described^7,8,40,41^. Briefly, cells were extracted with NETN300 buffer [20 mM Hepes (pH 7.5), 300 mM NaCl, and 0.5% NP-40] containing benzonase, protease, and phosphatase inhibitor cocktails (Roche), followed by incubation at 4 °C for 30 min. The insoluble fraction was removed by centrifugation, and the soluble fraction was immunoprecipitated with an anti-FLAG antibody. Precipitates were analyzed by Western blotting.

### Statistical analysis

All statistical tests are reported in each associated figure and figure legend. All statistical analyses were performed using GraphPad Prism (RRID:SCR_002798), with significance defined as *P* < 0.05.

### Reporting summary

Further information on research design is available in the Nature Portfolio Reporting Summary linked to this article.

## Supplementary information

Transparent Peer Review file

Supplementary Figs.

Description of Additional Supplementary Files

Supplementary data

Reporting Summary

## Data Availability

The data and supporting materials are available within the article and from its online Supplementary material. Uncropped western blot images are provided in Supplementary Fiures S8–S10. The source data behind the graphs can be found in the Supplementary Data file.
