# Supplementary material for: ISWI/CHRAC orchestrates sequential chromatin-remodeling activities by mobilizing DNA polymerase ε for homologous recombination
Source: Commun Biol. 2026 Sep 15;9:1177. doi: 10.1038/s42003-026-10862-0 (PMC13578426; doi:10.1038/s42003-026-10862-0)
Supplement: Supplementary file 1 [file 42003_2026_10862_MOESM1_ESM.pdf]

nature portfolio

Peer Review File

ISWI/CHRAC orchestrates sequential chromatin-remodeling activities by mobilizing DNA polymerase ε for homologous recombination

Corresponding Author: Dr Ayako Ui

This file contains all reviewer reports in order by version, followed by all author rebuttals in order by version.

Version 0:

Reviewer comments:

Reviewer #1

(Remarks to the Author)
This paper proposes that CHRAC17 promotes homologous recombination (HR) through its nucleosome assembly and sliding function, acting via ACF complex recruitment and POLE3 within DNA polymerase ε. The topic is novel and relevant to genome integrity research, as there has been no direct report on CHRAC17 function in DNA damage response. However, several major issues need to be addressed to substantiate the conclusions.

Major Comments

1. Evidence for nucleosome sliding and assembly at DSBs is lacking.
The title emphasizes "ISWI/CHRAC-driven nucleosome assembly and sliding," but there is no direct evidence at DSB sites. Please examine SNF2H localization (in addition to ACF1) and its dependency on CHRAC17. Is SNF2H recruitment affected in CHRAC17-depleted or N/C-terminal mutant cells? Compare SNF2H binding to CHRAC17 WT vs N-terminal mutant; do these differences correlate with the functional phenotypes (e.g., Fig. 3)? The authors also should clarify whether nucleosome sliding is required for the further progression of homologous recombination (HR). At least the evidence through protein interaction analysis should be provided to support this point.

2. Relationship with polymerase ε activity.
Clarify whether CHRAC17-mediated nucleosome remodeling affects Pol ε recruitment or activity during HR repair. This would connect CHRAC17's dual role as a remodeling subunit and as POLE3.

3. ATM regulation of CHRAC15–CHRAC17 interaction.
Since CHRAC17 recruitment is CHRAC15-dependent, test whether CHRAC15 is regulated by ATM kinase.
If ATM signaling plays a major role, the authors should clarify whether CHRAC15 binding or ATM-mediated activation is more critical for CHRAC17 recruitment and HR function. The use of ATM, ATR, and DNA-PK inhibitors as controls is recommended to demonstrate pathway specificity.

In addition, the authors cited Park et al., EMBO, 2006 to suggest that SWI/SNF activates ATM; however, that study shows that SWI/SNF directly binds to chromatin and modulates nucleosome structure rather than activating ATM signaling. Therefore, if CHRAC17 recruitment depends on the presence of BRM1, this would imply that its recruitment is influenced by chromatin dynamics regulated by SWI/SNF, rather than directly by ATM activation. Clarification of this distinction is necessary.

4. Include C-terminal mutant analysis.
In Figures 2–3, include C-terminal mutants alongside the N-terminal versions to show whether they fail to rescue HR defects.

Minor Comments

1.If RAD51 recruitment occurs but downstream signaling is impaired, analysis of downstream HR factors (e.g., RAD54, FANCD2) would strengthen the mechanistic link.
2. A neutral comet assay would help confirm DSB repair defects.
3. It would be valuable to include CHRAC17 recruitment analysis using a ChIP time-course experiment (in Figure 5H) to show early recruitment of CHRAC17 and late reruitment of Pol ε in following DNA damage.

Reviewer #2

(Remarks to the Author)

Ui et al.

ISWI/CHRAC drives nucleosome assembly and sliding by mobilizing DNA polymerase ε for homologous recombination

The H2B-type histone-fold protein CHRAC17 is an integral part of the chromatin remodeling complex CHRAC and a subunit (POLE3) of DNA polymerase epsilon, which is mainly responsible for leading strand DNA synthesis during replication. Chromatin poses a natural barrier to recombination at multiple stages including DSB resection and D-loop formation, whereas emerging evidence suggests that nucleosome assembly favors later stages of recombination. DNA polymerase epsilon has been implicated in homologous recombination in the budding yeast, likely contributing to gap filling in the second end synthesis step during SDSA, BIR or in the double-Holiday junction pathway. The manuscript documents several roles of CHRAC during recombination. CHRAC17 recruits the SNF2H-ACF1 complex to DSBs through its N-terminal H2B type histone-fold domain. This domain is sufficient for nucleosome assembly but incapable to support nucleosome sliding, and was found to support BRCA1 and RAD51 recruitment to DSBs. However, this domain did not support recombination activity measured by the DR-GFP reporter, suggesting a second role of CHRAC17 in recombination that may depend on nucleosome sliding. To study the role of POL epsilon, the author chose to focus on the non-essential subunit POLE4, as CHRAC17 (POLE3) has pleiotropic effects. POLE4 knockdown did not affect RAD51 focus formation after IR but slowed their turnover, suggesting a later role of POL epsilon consistent with a defect in the DR-GFP reporter assay and the increased PARPi sensitivity. Two independent approaches showed a late recruitment of POLE4 to DNA damage induced by laser or a single DSB by I-SceI which is consistent with a role in HR-associated DNA synthesis. In general, the results support the conclusions, and the experiments were conducted with high technical quality.

1) The CHRAC17 data using back-complementation are strong. Such back-complementation data are lacking for POLE4. Instead, two different siRNAs are used, which somewhat mitigates concerns about specificity. Validation of a key experiment such as the DR-GFP reporter assays in a back-complementation would significantly strengthen the point about the contribution of POLE4 and by extension POL epsilon in recombination.

2) It is surprising that the nucleosome assembly but not the nucleosome sliding activity of CHRAC is required for BRCA1 and RAD51 focus formation after IR. The naïve expectation would have been that nucleosome sliding is required for resection. Have the authors examined DSB resection under these conditions or at the DR-GFP reporter?

3) Figure 5: It is not clear what is shown in parts D and F. Please define which time point is shown.

4) Figure 5: The ChIP time course is conceptually an exciting experiment, emulating studies in yeast. However, the ChIP data show low levels (max. 0.0% of input). This low could be owed to the asynchrony of the system. Hence, additional controls which further validate that the POLE2 signal is real would greatly strengthen the argument. One possible control would be to show RAD51 dependence of the signal using RAD51 inhibitors and knockdown.

5) Please check Figure S3, panel C seems to be missing. What is labeled C is likely panel D.

Reviewer #3

(Remarks to the Author)
The manuscript proposes a two-phase model for CHRAC/ISWI action at DSBs: an initial nucleosome-assembly step that enables BRCA1/RAD51 loading, followed by nucleosome sliding that promotes RAD51-mediated steps. The authors further suggest that CHRAC17/POLE3 subsequently recruits the Polε complex to drive later stages of HR.

The manuscript is written in poor English. Numerous sentences are difficult to understand, and several conclusions are overstated because it is unclear what evidence they are based on. There are too many unclear passages to rewrite one by one; comprehensive language editing is needed.

Major comments
ATM-CHRAC17 causal link. The claim that ATM signaling “regulates” CHRAC17 accumulation rests solely on an ATM inhibitor reducing CHRAC17 recruitment (Fig. 1H), but appropriate controls are missing. The authors should add dose/chemotype controls for the inhibitor, target-engagement readouts (loss of pATM/pKAP1/pCHK2 under identical conditions), and genetic validation (ATM knockout and/or kinase-dead rescue). Consider ATR/DNA-PK controls to exclude off-target checkpoint effects.

The authors conclude that CHRAC17-N "is insufficient to complete HR," yet early BRCA1/RAD51 recruitment appears normal while γH2AX/RAD51 foci persist. They should operationalize "completion" (e.g., DR-GFP gene conversion, RAD51-foci resolution kinetics, D-loop extension/DNA-synthesis assays, junction sequencing) and provide data that directly mark these endpoints.

Rescue-experiment caveats (overexpression and mechanism). The CHRAC17-WT/-N rescues rely on overexpression, which may bypass stoichiometric constraints. The authors should include (i) expression-matched rescues titrated to near-endogenous levels, (ii) endogenous tagging or CRISPRi rescue if feasible, and (iii) cellular evidence that CHRAC17-N truly lacks sliding activity (not only in vitro precedent). Without this, the conclusion that "assembly without sliding loads but cannot progress HR" remains tentative.

The sentence "Pol ε does not influence recruitment, rather than stalling, of RAD51 at DSB sites, implying that RAD51-mediated recombination processes are disturbed" is unclear. If the intended meaning is that Pol ε does not alter initial RAD51 recruitment but its loss causes persistent RAD51 foci (i.e., stalling) and thus impairs downstream RAD51-dependent steps, the authors should provide evidence separating recruitment from progression (e.g., impaired D-loop extension, or decreased DNA synthesis within repair tracts).

The topic is interesting and potentially impactful, but key mechanistic claims require additional controls and experiments, as well as improved writing, before the conclusions can be fully supported.

Version 1:

Reviewer comments:

Reviewer #1

(Remarks to the Author)
I thank the authors for their careful revision. The authors have adequately addressed my comments, and the revised manuscript is now clearer and better supported. I have no further major concerns.

Reviewer #2

(Remarks to the Author)

Revised mansucript

The H2B-type histone-fold protein CHRAC17 is an integral part of the chromatin remodeling complex CHRAC and a subunit (POLE3) of DNA polymerase epsilon, which is mainly responsible for leading strand DNA synthesis during replication. Chromatin poses a natural barrier to recombination at multiple stages including DSB resection and D-loop formation, whereas emerging evidence suggests that nucleosome assembly favors later stages of recombination. DNA polymerase epsilon has been implicated in homologous recombination in the budding yeast, likely contributing to gap filling in the second end synthesis step during SDSA, BIR or in the double-Holiday junction pathway. The manuscript documents several roles of CHRAC during recombination. CHRAC17 recruits the SNF2H-ACF1 complex to DSBs through its N-terminal H2B type histone-fold domain. This domain is sufficient for nucleosome assembly but incapable to support nucleosome sliding, and was found to support BRCA1 and RAD51 recruitment to DSBs. However, this domain did not support recombination activity measured by the DR-GFP reporter, suggesting a second role of CHRAC17 in recombination that may depend on nucleosome sliding. To study the role of POL epsilon, the author chose to focus on the non-essential subunit POLE4, as CHRAC17 (POLE3) has pleiotropic effects. POLE4 knockdown did not affect RAD51 focus formation after IR but slowed their turnover, suggesting a later role of POL epsilon consistent with a defect in the DR-GFP reporter assay and the increased PARPi sensitivity. Two independent approaches showed a late recruitment of POLE4 to DNA damage induced by laser or a single DSB by I-SceI which is consistent with a role in HR-associated DNA synthesis. In general, the results support the conclusions, and the experiments were conducted with high technical quality.

The revisions nicely addresses the previous concerns, further improving an already strong contribution.

1) The CHRAC17 data using back-complementation are strong. Such back-complementation data are lacking for POLE4. Instead, two different siRNAs are used, which somewhat mitigates concerns about specificity. Validation of a key experiment such as the DR-GFP reporter assays in a back-complementation would significantly strengthen the point about the contribution of POLE4 and by extension POL epsilon in recombination.

Revision:
The addition of the backcomplementation in Figure S6A fully addresses this point.

2) It is surprising that the nucleosome assembly but not the nucleosome sliding activity of CHRAC is required for BRCA1 and RAD51 focus formation after IR. The naïve expectation would have been that nucleosome sliding is required for resection. Have the authors examined DSB resection under these conditions or at the DR-GFP reporter?

Revision:
The assay in Figure S4a to monitor resection is rather indirect, but I agree that the efficient recruitment of BRCA1 and RAD51 is consistent with normal resection.

3) Figure 5: It is not clear what is shown in parts D and F. Please define which time point is shown.

Revision:
OK

4) Figure 5: The ChIP time course is conceptually an exciting experiment, emulating studies in yeast. However, the ChIP data show low levels (max. 0.0% of input). This low could be owed to the asynchrony of the system. Hence, additional controls which further validate that the POLE2 signal is real would greatly strengthen the argument. One possible control would be to show RAD51 dependence of the signal using RAD51 inhibitors and knockdown.

Revision:
The data in Figure S7e nicely validate that the POLE ChIP signal depends on RAD51.

5) Please check Figure S3, panel C seems to be missing. What is labeled C is likely panel D.

Revision:
OK

Reviewer #3

(Remarks to the Author)
The revised manuscript has been substantially improved. The authors have added experiments, including analyses of SNF2H recruitment, CHRAC17–SNF2H interaction, POLE4 complementation, DNA-end resection, RAD54 recruitment, RAD51-dependent POLE2 recruitment, and a site-specific HR completion assay. These data address many of the previous concerns and strengthen the proposed role of CHRAC17/POLE3 in homologous recombination.
However, I still think that some conclusions should be phrased more cautiously. In particular, the manuscript continues to make strong statements about nucleosome sliding at DSBs, although direct evidence for nucleosome sliding in vivo is still lacking. The data support the idea that CHRAC17-N allows early HR factor recruitment but is insufficient for later HR steps, which is consistent with a requirement for an additional CHRAC remodeling activity. However, this should not be presented as direct evidence that nucleosome sliding occurs or is mechanistically responsible, unless more direct experimental support is provided. I therefore recommend that the authors soften the title, abstract, and relevant statements in the Results and Discussion. Phrases such as “CHRAC utilizes its nucleosome-sliding activity” could be replaced by more cautious wording, for example “CHRAC promotes later RAD51-mediated HR steps through a process consistent with its nucleosome-remodeling activity.” In addition, the limitations of the C-terminal mutant analysis should be stated more clearly, since the lack of detectable expression prevents a formal functional rescue experiment.

Version 2:

Reviewer comments:

Reviewer #3

(Remarks to the Author)
The authors have responded satisfactorily to my comments and have adequately addressed the issues raised in my review. I therefore have no further concerns and recommend that the manuscript be accepted for publication.

Open Access This Peer Review File is licensed under a Creative Commons Attribution 4.0 International License, which permits use, sharing, adaptation, distribution and reproduction in any medium or format, as long as you give appropriate credit to the original author(s) and the source, provide a link to the Creative Commons license, and indicate if changes were made.
In cases where reviewers are anonymous, credit should be given to 'Anonymous Referee' and the source.
The images or other third party material in this Peer Review File are included in the article's Creative Commons license, unless indicated otherwise in a credit line to the material. If material is not included in the article's Creative Commons license and your intended use is not permitted by statutory regulation or exceeds the permitted use, you will need to obtain permission directly from the copyright holder.
To view a copy of this license, visit https://creativecommons.org/licenses/by/4.0/

(Response to Reviewers' comments)

Reviewer #1 (Remarks to the Author):

This paper proposes that CHRAC17 promotes homologous recombination (HR) through its nucleosome assembly and sliding function, acting via ACF complex recruitment and POLE3 within DNA polymerase ε. The topic is novel and relevant to genome integrity research, as there has been no direct report on CHRAC17 function in DNA damage response. However, several major issues need to be addressed to substantiate the conclusions.

We thank the reviewers for their constructive suggestions and thoughtful comments. The reviewers' comments are presented in a numbered format, and our point-by-point responses are provided under each corresponding comment.

Following the instructions, for all graphs depicting a single point value (e.g., mean) with error bars, we have added individual data points or converted the graphs to boxplots or dot plots to better illustrate data distribution.

Major Comments

1. Evidence for nucleosome sliding and assembly at DSBs is lacking.
The title emphasizes "ISWI/CHRAC-driven nucleosome assembly and sliding," but there is no direct evidence at DSB sites. Please examine SNF2H localization (in addition to ACF1) and its dependency on CHRAC17. Is SNF2H recruitment affected in CHRAC17-depleted or N/C-terminal mutant cells? Compare SNF2H binding to CHRAC17 WT vs N-terminal mutant; do these differences correlate with the functional phenotypes (e.g., Fig. 3)? The authors also should clarify whether nucleosome sliding is required for the further progression of homologous recombination (HR). At least the evidence through protein interaction analysis should be provided to support this point.

We thank the reviewer for this constructive comment and agree that additional experiments are required to address whether SNF2H recruitment is affected in CHRAC17-depleted or N/C-terminal mutant cells, and whether differences in SNF2H binding between CHRAC17 WT and the N-terminal mutant correlate with the observed functional phenotypes.

In response, we performed micro-laser irradiation assays and found that CHRAC17 is required for efficient SNF2H recruitment to DSB sites (Supplementary Fig. S1b), indicating that CHRAC17 is necessary for SNF2H recruitment in vivo.

We next examined whether the CHRAC17 N-terminal mutant affects interaction with SNF2H. Immunoprecipitation analyses showed that CHRAC17-N, like CHRAC17-WT, retains the ability to interact with SNF2H (Supplementary Fig. S2c), consistent with previous reports that CHRAC17-N can interact with ACF1 [1], which forms a complex with SNF2H.

The new data have been included in Supplementary Fig. S1b and S2c and are described in the manuscript (page 5 line 24 and page 7 lines 9).

2. Relationship with polymerase ε activity.
Clarify whether CHRAC17-mediated nucleosome remodeling affects Pol ε recruitment or activity during HR repair. This would connect CHRAC17's dual role as a remodeling subunit and as POLE3.

We thank the reviewer for this insightful comment. To address the reviewer's comment, we examined whether CHRAC17-N promotes recruitment of Pol ε to DSB sites. As shown in Supplementary Fig. S7f, CHRAC17-WT efficiently supported Pol ε accumulation at DSBs, whereas CHRAC17-N failed to do so. These results indicate that CHRAC17-dependent nucleosome remodeling is required for Pol ε recruitment during HR repair.

The new data have been included in Supplementary Fig. S7f and are described in the manuscript (page 11 lines 8 and page 14 lines 26).

3. ATM regulation of CHRAC15–CHRAC17 interaction.
Since CHRAC17 recruitment is CHRAC15-dependent, test whether CHRAC15 is regulated by ATM kinase.
If ATM signaling plays a major role, the authors should clarify whether CHRAC15 binding or ATM-mediated activation is more critical for CHRAC17 recruitment and HR function. The use of ATM, ATR, and DNA-PK inhibitors as controls is recommended to demonstrate pathway specificity.

We thank the reviewer for this important suggestion. We previously showed that CHRAC17 recruitment is CHRAC15-dependent and therefore examined CHRAC15 localization at DSB sites. As shown in Supplementary Fig. S1a, CHRAC15 recruitment was detectable but weak, preventing clear assessment of its dependence on ATM, ATR, or DNA-PKcs.

We next examined whether CHRAC17 recruitment depends on ATM, ATR, or DNA-PKcs activity. As shown in Supplementary Fig. S1f, CHRAC17 accumulation at DSB sites was reduced by ATM inhibition and CHRAC15 knockdown, but was unaffected by ATR or DNA-PKcs inhibition. These results indicate that CHRAC15 and ATM signaling are required for

efficient CHRAC17 recruitment to DNA damage sites.

The new data have been included in Supplementary Fig. S1a and S1f and are described in the manuscript (page 5 line 11, page 6 lines 23 and page 12 line 30).

4. In addition, the authors cited Park et al., EMBO, 2006 to suggest that SWI/SNF activates ATM; however, that study shows that SWI/SNF directly binds to chromatin and modulates nucleosome structure rather than activating ATM signaling. Therefore, if CHRAC17 recruitment depends on the presence of BRM1, this would imply that its recruitment is influenced by chromatin dynamics regulated by SWI/SNF, rather than directly by ATM activation. Clarification of this distinction is necessary.

We thank the reviewer for this important and insightful comment. We apologize for the incorrect citation and have now corrected it. Lee et al. (2010) suggested that The SWI/SNF catalytic subunits BRG1 and BRM have been shown to interact with nucleosomes containing γH2AX and acetylated H3[2].The previous studies have proposed that recruited SWI/SNF facilitates ATM-mediated H2AX S139 phosphorylation, likely by increasing nucleosome accessibility around DSBs [2] and also promotes DSB repair [3]. We found that CHRAC17 recruitment to DSB sites requires BRM, a catalytic subunit of the SWI/SNF complex (Supplementary Fig. S1e), suggesting that SWI/SNF-mediated chromatin remodeling plays an important role in CHRAC17 recruitment at DSB sites. In addition, we found that inhibition of ATM signaling reduced CHRAC17 recruitment to DSB sites (Fig. 1h). Together, these findings suggest that SWI/SNF-mediated chromatin remodeling likely facilitates CHRAC17 recruitment but is not sufficient in the absence of ATM signaling.

We corrected the comments in the manuscript (page 6 lines 18 and page 12 lines 23).

5. Include C-terminal mutant analysis.
In Figures 2–3, include C-terminal mutants alongside the N-terminal versions to show whether they fail to rescue HR defects.

We thank the reviewer for this helpful suggestion. As recommended, we first generated a T-REx–CHRAC17 C-terminal mutant plasmid designed for doxycycline-inducible expression. We constructed two independent plasmids, verified their sequences, and transfected them into T-REx 293 cells (Thermo Fisher Scientific), but we were unable to detect expression of the CHRAC17 C-terminal mutant protein, although the CHRAC17-WT plasmid was readily expressed (following, Figure A).

We next generated a Flp-In T-REx–CHRAC17 C-terminal mutant plasmid for stable

expression under the CMV promoter. Again, we constructed two independent plasmids, verified their sequences, and transfected them into T-REx 293 cells, but we could not detect mutant protein expression, whereas CHRAC17-WT was readily expressed (in the following Figure A).

We also attempted to generate stable T-REx cell lines on three separate occasions, but still did not detect mutant protein expression (in the following Figure B).

Given its small size (~8 kDa), the CHRAC17 C-terminal mutant protein may be unstable, difficult to express, or toxic to cells. Although we could not assess its phenotype due to lack of expression, it failed to accumulate at DSB sites, suggesting it is unlikely to function in DSB repair.

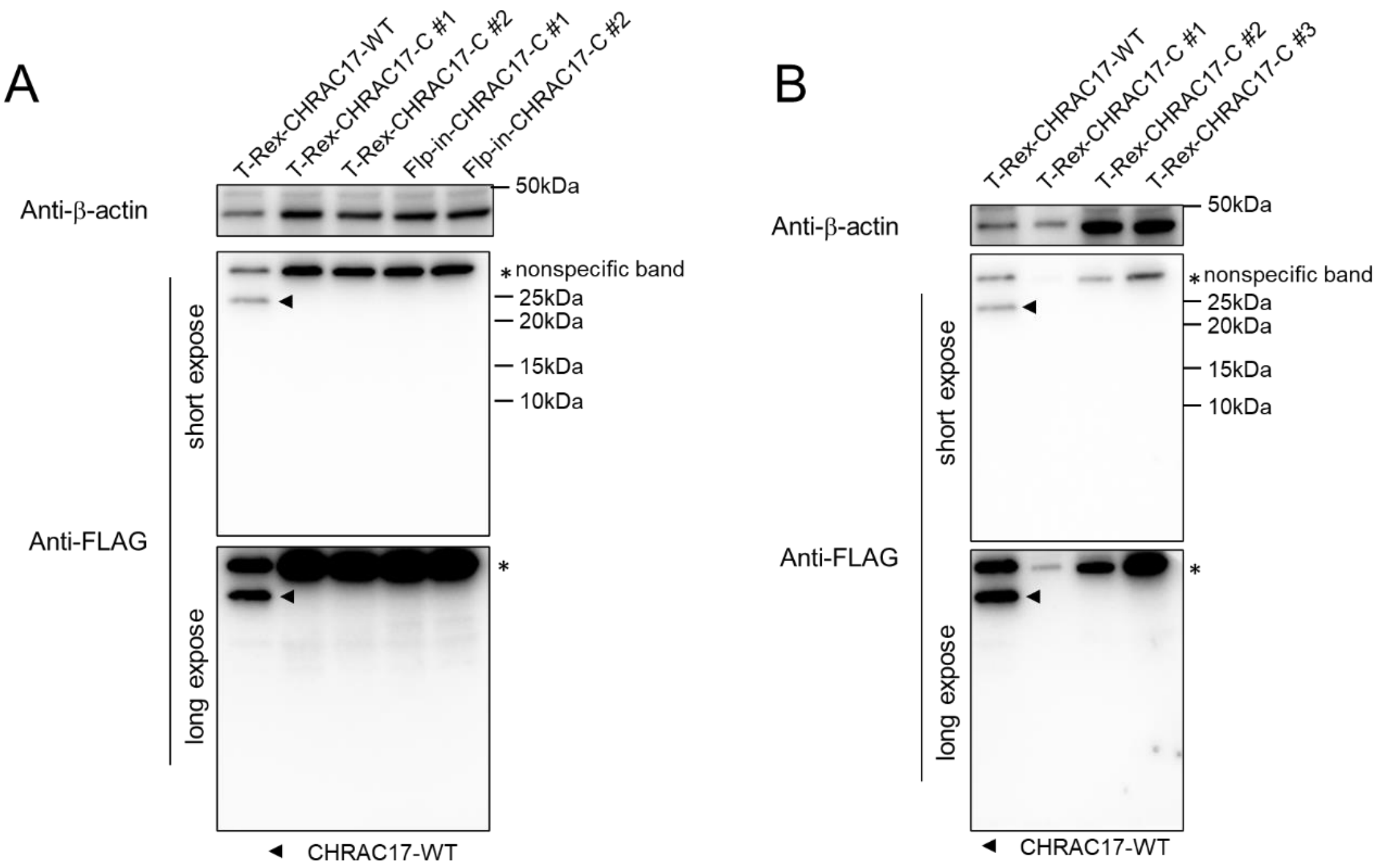

Minor Comments

1.If RAD51 recruitment occurs but downstream signaling is impaired, analysis of downstream HR factors (e.g., RAD54, FANCD2) would strengthen the mechanistic link.

We thank the reviewer for this valuable suggestion and agree that analysis of downstream HR factors would strengthen the mechanistic interpretation.

In response, we examined the recruitment of RAD54, a key downstream effector of RAD51, in cells expressing the CHRAC17-N mutants. As shown in Supplementary Fig. S4b and S4c, RAD54 recruitment to DSB sites was observed in CHRAC17-N cells, comparable to that in

CHRAC17-WT cells.

The new data have been included in Supplementary Fig.S4b and S4c and are described in the manuscript (page 8 lines 10 and page 13 lines 7).

2. A neutral comet assay would help confirm DSB repair defects.

We thank the reviewer for this helpful suggestion. As we did not have access to the equipment required to perform a comet assay, we addressed this point by applying an independent, quantitative, site-specific DSB repair assay (Supplementary Fig. S5a) 4. In this assay, DSBs are introduced at a defined genomic locus using CRISPR/Cas9, and BRCA1- and RAD51-mediated HR are quantified by real-time PCR with locus-specific primers, providing a direct measure of DSB repair efficiency. As shown in Supplementary Fig. S5b, CHRAC17 knockdown significantly reduced DSB repair, whereas re-expression of CHRAC17-WT restored repair activity. In contrast, neither CHRAC17-N mutant rescued the DSB repair defect relative to CHRAC17-WT.

The new data have been included in Supplementary Fig.S5a and S5b and are described in the manuscript section (page 9 lines 11 and page 13 lines 7).

3. It would be valuable to include CHRAC17 recruitment analysis using a ChIP time-course experiment (in Figure 5H) to show early recruitment of CHRAC17 and late reruitment of Pol ε in following DNA damage.

We further performed a ChIP time-course analysis to examine CHRAC17 recruitment to DSB sites following DNA damage (Supplementary Fig. S7d). CHRAC17 was rapidly recruited after DSB induction, with kinetics similar to γ-H2AX, as determined by ChIP analysis, consistent with an early role in repair. CHRAC17 recruitment was sustained after DSB induction and further increased at time points when POLE2 recruitment was detected by ChIP analysis.

The new data have been included in Supplementary Fig.S7d and are described in the manuscript (page 11 lines 4).

Reviewer #2 (Remarks to the Author):

Ui et al.

ISWI/CHRAC drives nucleosome assembly and sliding by mobilizing DNA polymerase ε for homologous recombination

The H2B-type histone-fold protein CHRAC17 is an integral part of the chromatin remodeling complex CHRAC and a subunit (POLE3) of DNA polymerase epsilon, which is mainly responsible for leading strand DNA synthesis during replication. Chromatin poses a natural barrier to recombination at multiple stages including DSB resection and D-loop formation, whereas emerging evidence suggests that nucleosome assembly favors later stages of recombination. DNA polymerase epsilon has been implicated in homologous recombination in the budding yeast, likely contributing to gap filling in the second end synthesis step during SDSA, BIR or in the double-Holiday junction pathway. The manuscript documents several roles of CHRAC during recombination. CHRAC17 recruits the SNF2H-ACF1 complex to DSBs through its N-terminal H2B type histone-fold domain. This domain is sufficient for nucleosome assembly but incapable to support nucleosome sliding, and was found to support BRCA1 and RAD51 recruitment to DSBs. However, this domain did not support recombination activity measured by the DR-GFP reporter, suggesting a second role of CHRAC17 in recombination that may depend on nucleosome sliding. To study the role of POL epsilon, the author chose to focus on the non-essential subunit POLE4, as CHRAC17 (POLE3) has pleiotropic effects. POLE4 knockdown did not affect RAD51 focus formation after IR but slowed their turnover, suggesting a later role of POL epsilon consistent with a defect in the DR-GFP reporter assay and the increased PARPi sensitivity. Two independent approaches showed a late recruitment of POLE4 to DNA damage induced by laser or a single DSB by I-SceI which is consistent with a role in HR-associated DNA synthesis. In general, the results support the conclusions, and the experiments were conducted with high technical quality.

We thank the reviewers for their constructive suggestions and thoughtful comments. The reviewers' comments are presented in a numbered format, and our point-by-point responses are provided under each corresponding comment.

Following the instructions, for all graphs depicting a single point value (e.g., mean) with error bars, we have added individual data points or converted the graphs to boxplots or dot plots to better illustrate data distribution.

(1) The CHRAC17 data using back-complementation are strong. Such back-complementation data are lacking for POLE4. Instead, two different siRNAs are used, which somewhat mitigates concerns about specificity. Validation of a key experiment such as the DR-GFP reporter assays in a back-complementation would significantly strengthen the point about the contribution of POLE4 and by extension POL epsilon in recombination.

We thank the reviewer for this helpful suggestion. Following this recommendation, we generated a POLE4 expression plasmid and performed a complementation assay using an siRNA targeting the untranslated region of the POLE4 transcript (siPOLE4#2). As shown in Supplementary Fig.S6A, POLE4 re-expression restored homologous recombination activity in the DR-GFP reporter assay.

The new data have been included in Supplementary Fig.S6a and are described in manuscript (page 10 lines 2).

2) It is surprising that the nucleosome assembly but not the nucleosome sliding activity of CHRAC is required for BRCA1 and RAD51 focus formation after IR. The naïve expectation would have been that nucleosome sliding is required for resection. Have the authors examined DSB resection under these conditions or at the DR-GFP reporter?

We thank the reviewer for this insightful comment. In response to this suggestion, we examined DNA-end resection in cells expressing CHRAC17 mutant proteins. As shown in Supplementary Fig. S4a, DNA-end resection, as assessed by RPA phosphorylation, was not impaired in CHRAC17-N mutant cells and was comparable to that observed in CHRAC17-WT cells. These results are consistent with the efficient recruitment of BRCA1 and RAD51 upon expression of CHRAC17-WT and -N.

These results have been added as Supplementary Fig.S4a and are described in the manuscript (page 8 lines 8 and page 13 line 7).

3) Figure 5: It is not clear what is shown in parts D and F. Please define which time point is shown.

We thank the reviewer for this helpful comment. We apologize for the lack of clarity in the original figure. We have now clearly defined the time points in panels 5d and 5f in both figures and corresponding figure legend.

4) Figure 5: The ChIP time course is conceptually an exciting experiment, emulating studies in yeast. However, the ChIP data show low levels (max. 0.0% of input). This low could be owed to the asynchrony of the system. Hence, additional controls which further validate that the POLE2 signal is real would greatly strengthen the argument. One possible control would be to show RAD51 dependence of the signal using RAD51 inhibitors and knockdown.

We thank the reviewer for this constructive comment. To further validate that the POLE2

ChIP signal reflects specific recruitment to DSB sites, we examined whether POLE2 recruitment depends on key homologous recombination factor, RAD51. We analyzed the effect of RAD51 depletion on POLE2 enrichment at DSB sites. As shown in Supplementary Fig.S7E, RAD51 knockdown reduced POLE2 recruitment.

These data have been added as Supplementary Fig.S7e and are described in the manuscript (page 11 lines 5).

5) Please check Figure S3, panel C seems to be missing. What is labeled C is likely panel D.

We thank the reviewer for carefully checking the manuscript and for pointing out this labeling error. We have edited and corrected the Supplementary Figures.

Reviewer #3 (Remarks to the Author):

The manuscript proposes a two-phase model for CHRAC/ISWI action at DSBs: an initial nucleosome-assembly step that enables BRCA1/RAD51 loading, followed by nucleosome sliding that promotes RAD51-mediated steps. The authors further suggest that CHRAC17/POLE3 subsequently recruits the Polε complex to drive later stages of HR.

The manuscript is written in poor English. Numerous sentences are difficult to understand, and several conclusions are overstated because it is unclear what evidence they are based on. There are too many unclear passages to rewrite one by one; comprehensive language editing is needed.

We thank the reviewers for their constructive suggestions and thoughtful comments. The reviewers' comments are presented in a numbered format, and our point-by-point responses are provided under each corresponding comment.

Following the instructions, for all graphs depicting a single point value (e.g., mean) with error bars, we have added individual data points or converted the graphs to boxplots or dot plots to better illustrate data distribution.

Major comments

1. ATM-CHRAC17 causal link. The claim that ATM signaling "regulates" CHRAC17 accumulation rests solely on an ATM inhibitor reducing CHRAC17 recruitment (Fig. 1H), but appropriate controls are missing. The authors should add dose/chemotype controls for the inhibitor, target-engagement readouts (loss of pATM/pKAP1/pCHK2 under identical conditions), and genetic validation (ATM knockout and/or kinase-dead rescue). Consider

ATR/DNA-PK controls to exclude off-target checkpoint effects.

We thank the reviewer for this important comment. We assessed ATM inhibition by examining phosphorylation of ATM and CHK2 after IR treatment. As shown in Supplementary Fig. S2a, the ATM inhibitor reduced ATM and CHK2 phosphorylation following IR-induced DSBs. Consistently, ATM knockdown also decreased ATM and CHK2 phosphorylation after IR (Supplementary Fig. S2b), supporting the inhibitor-based results.

We further examined whether inhibition of ATM, ATR, or DNA-PKcs affects CHRAC17 recruitment to DSB sites. As shown in Supplementary Fig. S1f, GFP-CHRAC17 recruitment was reduced by ATM inhibition, whereas ATR or DNA-PKcs inhibition had no effect.

The new data have been included in Supplementary Fig. S1f, S2a and S2b are described in the manuscript (page 6 lines 23, page 6 lines 25 and page 13 line 29).

2. The authors conclude that CHRAC17-N "is insufficient to complete HR," yet early BRCA1/RAD51 recruitment appears normal while γH2AX/RAD51 foci persist. They should operationalize "completion" (e.g., DR-GFP gene conversion, RAD51-foci resolution kinetics, D-loop extension/DNA-synthesis assays, junction sequencing) and provide data that directly mark these endpoints.

We thank the reviewer for this helpful suggestion. Recombination assay was performed (Supplementary Fig.S5a) [4], and DSBs are introduced at a defined genomic locus using CRISPR/Cas9, and BRCA1- and RAD51-mediated HR are quantified by real-time PCR with locus-specific primers, providing a direct measure of DSB repair efficiency. This assay directly measures the completion of the HR process at the level of DNA repair product formation (Supplementary Fig.S5a). As shown in Supplementary Fig.S5b, cells expressing CHRAC17-N had a significant defect in HR completion compared with cells expressing CHRAC17-WT.

The results have been added as Supplementary Fig.S5a and Fig.S5b, and are described in manuscript (page 9 lines 11).

3. Rescue-experiment caveats (overexpression and mechanism). The CHRAC17-WT/-N rescues rely on overexpression, which may bypass stoichiometric constraints. The authors should include (i) expression-matched rescues titrated to near-endogenous levels, (ii) endogenous tagging or CRISPRi rescue if feasible, and (iii) cellular evidence that CHRAC17-N truly lacks sliding activity (not only in vitro precedent). Without this, the conclusion that "assembly without sliding loads but cannot progress HR" remains tentative.

We sincerely thank the reviewer for these important and thoughtful comments regarding the limitations of our rescue experiments. We address each point below.

(i) Expression levels: We agree that expression-matched rescue at near-endogenous levels would be ideal. While precise titration to endogenous levels is technically challenging, we carefully assessed CHRAC17 overexpression effects. Notably, CHRAC17-N did not show additional or aberrant phenotypes compared with CHRAC17-WT in HR and DSB repair assays, suggesting no detectable overexpression artifacts.

(ii) Endogenous tagging or CRISPR-based rescue: We considered this strategy; however, CHRAC17-N lacks the C-terminal region and is mislocalized outside the nucleus (Supplementary Fig. S1d), indicating it requires an additional NLS for function in DSB repair. Thus, we considered that generating these cell lines (Ve, WT, and N) and repeating all corresponding rescue experiments would be technically challenging and beyond the scope of the current revision. Therefore, we adopted an expression-based rescue approach as a practical alternative.

(iii) Nucleosome sliding activity: We also considered that direct in vivo assessment is technically challenging and beyond the scope of this revision. We have therefore removed the claims that CHRAC17-N is defective in nucleosome sliding in cells from the Results section and now discuss this possibility only in the Discussion section.

4. The sentence "Pol ε does not influence recruitment, rather than stalling, of RAD51 at DSB sites, implying that RAD51-mediated recombination processes are disturbed" is unclear. If the intended meaning is that Pol ε does not alter initial RAD51 recruitment but its loss causes persistent RAD51 foci (i.e., stalling) and thus impairs downstream RAD51-dependent steps, the authors should provide evidence separating recruitment from progression (e.g., impaired D-loop extension, or decreased DNA synthesis within repair tracts).

We thank the reviewer for this important comment. Although direct measurement of D-loop extension is technically difficult within the revision period, we examined the temporal relationship between RAD51 and Pol ε during HR. ChIP analysis showed that RAD51 knockdown reduced Pol ε recruitment to DSB sites (Supplementary Fig. S7e), indicating that Pol ε acts downstream of RAD51. In addition, we performed a site-specific homologous recombination assay (Supplementary Fig. S5a) 4. In this assay, DSBs are introduced at a defined genomic locus using CRISPR/Cas9, and BRCA1- and RAD51-mediated HR are quantified by real-time PCR with locus-specific primers, providing a direct measure of repair completion. As shown in Supplementary Fig. S6b, POLE4 knockdown significantly impaired HR completion

despite normal RAD51 focus formation, supporting a role for Pol ε in late HR steps after RAD51-mediated intermediates form.
The newly added data and textual changes are shown in Supplementary S5a, S6b and S7e, and described in the manuscript (page 10 lines 8, page 11 lines 5 and page 14 line 27).

5. The topic is interesting and potentially impactful, but key mechanistic claims require additional controls and experiments, as well as improved writing, before the conclusions can be fully supported.

We sincerely thank the reviewer for this constructive assessment of our work. We believe that these revisions have improved the manuscript and strengthened the support for our conclusions.

(References)

1 Kukimoto, I., Elderkin, S., Grimaldi, M., Oelgeschlager, T. & Varga-Weisz, P. D. The histone-fold protein complex CHRAC-15/17 enhances nucleosome sliding and assembly mediated by ACF. *Molecular Cell* **13**, 265-277 (2004). https://doi.org:Doi 10.1016/S1097-2765(03)00523-9

2 Lee, H. S., Park, J. H., Kim, S. J., Kwon, S. J. & Kwon, J. A cooperative activation loop among SWI/SNF, gamma-H2AX and H3 acetylation for DNA double-strand break repair. *The EMBO journal* **29**, 1434-1445 (2010). https://doi.org:10.1038/emboj.2010.27

3 Park, J. H. *et al.* Mammalian SWI/SNF complexes facilitate DNA double-strand break repair by promoting gamma-H2AX induction. *The EMBO journal* **25**, 3986-3997 (2006). https://doi.org:10.1038/sj.emboj.7601291

4 Yoshino, Y. *et al.* Evaluation of site-specific homologous recombination activity of BRCA1 by direct quantitation of gene editing efficiency. *Sci Rep-Uk* **9** (2019). https://doi.org:ARTN 1644
10.1038/s41598-018-38311-x

(Response to Reviewers' comments)

Reviewer #1 (Remarks to the Author):

I thank the authors for their careful revision. The authors have adequately addressed my comments, and the revised manuscript is now clearer and better supported. I have no further major concerns.

We sincerely thank the reviewer for the thoughtful and constructive comments and suggestions, which have greatly improved the quality and clarity of our manuscript.

Reviewer #2 (Remarks to the Author):

The H2B-type histone-fold protein CHRAC17 is an integral part of the chromatin remodeling complex CHRAC and a subunit (POLE3) of DNA polymerase epsilon, which is mainly responsible for leading strand DNA synthesis during replication. Chromatin poses a natural barrier to recombination at multiple stages including DSB resection and D-loop formation, whereas emerging evidence suggests that nucleosome assembly favors later stages of recombination. DNA polymerase epsilon has been implicated in homologous recombination in the budding yeast, likely contributing to gap filling in the second end synthesis step during SDSA, BIR or in the double-Holiday junction pathway. The manuscript documents several roles of CHRAC during recombination. CHRAC17 recruits the SNF2H-ACF1 complex to DSBs through its N-terminal H2B type histone-fold domain. This domain is sufficient for nucleosome assembly but incapable to support nucleosome sliding, and was found to support BRCA1 and RAD51 recruitment to DSBs. However, this domain did not support recombination activity measured by the DR-GFP reporter, suggesting a second role of CHRAC17 in recombination that may depend on nucleosome sliding. To study the role of POL epsilon, the author chose to focus on the non-essential subunit POLE4, as CHRAC17 (POLE3) has pleiotropic effects. POLE4 knockdown did not affect RAD51 focus formation after IR but slowed their turnover, suggesting a later role of POL epsilon consistent with a defect in the DR-GFP reporter assay and the increased PARPi sensitivity. Two independent approaches showed a late recruitment of POLE4 to DNA damage induced by laser or a single DSB by I-SceI which is consistent with a role in HR-associated DNA synthesis. In general, the results support the conclusions, and the experiments were conducted with high technical quality.

The revisions nicely addresses the previous concerns, further improving an already strong contribution.

1) The CHRAC17 data using back-complementation are strong. Such back-complementation data are lacking for POLE4. Instead, two different siRNAs are used, which somewhat mitigates concerns about specificity. Validation of a key experiment such as the DR-GFP reporter assays in a back-complementation would significantly strengthen the point about the contribution of POLE4 and by extension POL epsilon in recombination.

Revision:
The addition of the backcomplementation in Figure S6A fully addresses this point.

2) It is surprising that the nucleosome assembly but not the nucleosome sliding activity of CHRAC is required for BRCA1 and RAD51 focus formation after IR. The naïve expectation would have been that nucleosome sliding is required for resection. Have the authors examined DSB resection under these conditions or at the DR-GFP reporter?

Revision:
The assay in Figure S4a to monitor resection is rather indirect, but I agree that the efficient recruitment of BRCA1 and RAD51 is consistent with normal resection.

3) Figure 5: It is not clear what is shown in parts D and F. Please define which time point is shown.

Revision:
OK

4) Figure 5: The ChIP time course is conceptually an exciting experiment, emulating studies in yeast. However, the ChIP data show low levels (max. 0.0% of input). This low could be owed to the asynchrony of the system. Hence, additional controls which further validate that the POLE2 signal is real would greatly strengthen the argument. One possible control would be to show RAD51 dependence of the signal using RAD51 inhibitors and knockdown.

Revision:
The data in Figure S7e nicely validate that the POLE ChIP signal depends on RAD51.

5) Please check Figure S3, panel C seems to be missing. What is labeled C is likely panel D.

Revision:
OK

We sincerely thank the reviewer for the thoughtful and constructive comments and suggestions, which have greatly improved the quality and clarity of our manuscript.

Reviewer #3 (Remarks to the Author):

The revised manuscript has been substantially improved. The authors have added experiments, including analyses of SNF2H recruitment, CHRAC17–SNF2H interaction, POLE4 complementation, DNA-end resection, RAD54 recruitment, RAD51-dependent POLE2 recruitment, and a site-specific HR completion assay. These data address many of the previous concerns and strengthen the proposed role of CHRAC17/POLE3 in homologous recombination. However, I still think that some conclusions should be phrased more cautiously. In particular, the manuscript continues to make strong statements about nucleosome sliding at DSBs, although direct evidence for nucleosome sliding in vivo is still lacking. The data support the idea that CHRAC17-N allows early HR factor recruitment but is insufficient for later HR steps, which is consistent with a requirement for an additional CHRAC remodeling activity.

However, this should not be presented as direct evidence that nucleosome sliding occurs or is mechanistically responsible, unless more direct experimental support is provided. I therefore recommend that the authors soften the title, abstract, and relevant statements in the Results and Discussion. Phrases such as “CHRAC utilizes its nucleosome-sliding activity” could be replaced by more cautious wording, for example “CHRAC promotes later RAD51-mediated HR steps through a process consistent with its nucleosome-remodeling activity.”

We sincerely thank the reviewer for this important and insightful comment. Accordingly, we have carefully revised the title, abstract, and relevant statements throughout the Results and Discussion sections to adopt more cautious and appropriately qualified wording.

In addition, the limitations of the C-terminal mutant analysis should be stated more clearly, since the lack of detectable expression prevents a formal functional rescue experiment.

We thank the reviewer for this important comment. In response, we included the relevant experimental data in Supplementary Fig. S4d and S4e. In addition, we have described the limitations of the C-terminal mutant analysis in the revised manuscript (page 8 line 15).

We sincerely thank the reviewer for the thoughtful and constructive comments and suggestions, which have greatly improved the quality and clarity of our manuscript.

(Response to Reviewers' comments)

Reviewer #1 (Remarks to the Author):

I thank the authors for their careful revision. The authors have adequately addressed my comments, and the revised manuscript is now clearer and better supported. I have no further major concerns.

We sincerely thank the reviewer for the thoughtful and constructive comments and suggestions, which have greatly improved the quality and clarity of our manuscript.

Reviewer #2 (Remarks to the Author):

The H2B-type histone-fold protein CHRAC17 is an integral part of the chromatin remodeling complex CHRAC and a subunit (POLE3) of DNA polymerase epsilon, which is mainly responsible for leading strand DNA synthesis during replication. Chromatin poses a natural barrier to recombination at multiple stages including DSB resection and D-loop formation, whereas emerging evidence suggests that nucleosome assembly favors later stages of recombination. DNA polymerase epsilon has been implicated in homologous recombination in the budding yeast, likely contributing to gap filling in the second end synthesis step during SDSA, BIR or in the double-Holiday junction pathway. The manuscript documents several roles of CHRAC during recombination. CHRAC17 recruits the SNF2H-ACF1 complex to DSBs through its N-terminal H2B type histone-fold domain. This domain is sufficient for nucleosome assembly but incapable to support nucleosome sliding, and was found to support BRCA1 and RAD51 recruitment to DSBs. However, this domain did not support recombination activity measured by the DR-GFP reporter, suggesting a second role of CHRAC17 in recombination that may depend on nucleosome sliding. To study the role of POL epsilon, the author chose to focus on the non-essential subunit POLE4, as CHRAC17 (POLE3) has pleiotropic effects. POLE4 knockdown did not affect RAD51 focus formation after IR but slowed their turnover, suggesting a later role of POL epsilon consistent with a defect in the DR-GFP reporter assay and the increased PARPi sensitivity. Two independent approaches showed a late recruitment of POLE4 to DNA damage induced by laser or a single DSB by I-SceI which is consistent with a role in HR-associated DNA synthesis. In general, the results support the conclusions, and the experiments were conducted with high technical quality.

The revisions nicely addresses the previous concerns, further improving an already strong contribution.

1) The CHRAC17 data using back-complementation are strong. Such back-complementation data are lacking for POLE4. Instead, two different siRNAs are used, which somewhat mitigates concerns about specificity. Validation of a key experiment such as the DR-GFP reporter assays in a back-complementation would significantly strengthen the point about the contribution of POLE4 and by extension POL epsilon in recombination.

Revision:
The addition of the backcomplementation in Figure S6A fully addresses this point.

2) It is surprising that the nucleosome assembly but not the nucleosome sliding activity of CHRAC is required for BRCA1 and RAD51 focus formation after IR. The naïve expectation would have been that nucleosome sliding is required for resection. Have the authors examined DSB resection under these conditions or at the DR-GFP reporter?

Revision:
The assay in Figure S4a to monitor resection is rather indirect, but I agree that the efficient recruitment of BRCA1 and RAD51 is consistent with normal resection.

3) Figure 5: It is not clear what is shown in parts D and F. Please define which time point is shown.

Revision:
OK

4) Figure 5: The ChIP time course is conceptually an exciting experiment, emulating studies in yeast. However, the ChIP data show low levels (max. 0.0% of input). This low could be owed to the asynchrony of the system. Hence, additional controls which further validate that the POLE2 signal is real would greatly strengthen the argument. One possible control would be to show RAD51 dependence of the signal using RAD51 inhibitors and knockdown.

Revision:
The data in Figure S7e nicely validate that the POLE ChIP signal depends on RAD51.

5) Please check Figure S3, panel C seems to be missing. What is labeled C is likely panel D.

Revision:
OK

We sincerely thank the reviewer for the thoughtful and constructive comments and suggestions, which have greatly improved the quality and clarity of our manuscript.

Reviewer #3 (Remarks to the Author):

The revised manuscript has been substantially improved. The authors have added experiments, including analyses of SNF2H recruitment, CHRAC17–SNF2H interaction, POLE4 complementation, DNA-end resection, RAD54 recruitment, RAD51-dependent POLE2 recruitment, and a site-specific HR completion assay. These data address many of the previous concerns and strengthen the proposed role of CHRAC17/POLE3 in homologous recombination. However, I still think that some conclusions should be phrased more cautiously. In particular, the manuscript continues to make strong statements about nucleosome sliding at DSBs, although direct evidence for nucleosome sliding in vivo is still lacking. The data support the idea that CHRAC17-N allows early HR factor recruitment but is insufficient for later HR steps, which is consistent with a requirement for an additional CHRAC remodeling activity.

However, this should not be presented as direct evidence that nucleosome sliding occurs or is mechanistically responsible, unless more direct experimental support is provided. I therefore recommend that the authors soften the title, abstract, and relevant statements in the Results and Discussion. Phrases such as “CHRAC utilizes its nucleosome-sliding activity” could be replaced by more cautious wording, for example “CHRAC promotes later RAD51-mediated HR steps through a process consistent with its nucleosome-remodeling activity.”

We sincerely thank the reviewer for this important and insightful comment. Accordingly, we have carefully revised the title, abstract, and relevant statements throughout the Results and Discussion sections to adopt more cautious and appropriately qualified wording.

In addition, the limitations of the C-terminal mutant analysis should be stated more clearly, since the lack of detectable expression prevents a formal functional rescue experiment.

We thank the reviewer for this important comment. In response, we included the relevant experimental data in Supplementary Fig. S4d and S4e. In addition, we have described the limitations of the C-terminal mutant analysis in the revised manuscript (page 8 line 15).

We sincerely thank the reviewer for the thoughtful and constructive comments and suggestions, which have greatly improved the quality and clarity of our manuscript.
