# Supplementary material for: ISWI/CHRAC orchestrates sequential chromatin-remodeling activities by mobilizing DNA polymerase ε for homologous recombination
Source: Commun Biol. 2026 Sep 15;9:1177. doi: 10.1038/s42003-026-10862-0 (PMC13578426; doi:10.1038/s42003-026-10862-0)
Supplement: Supplementary file 2 [file 42003_2026_10862_MOESM2_ESM.pdf]

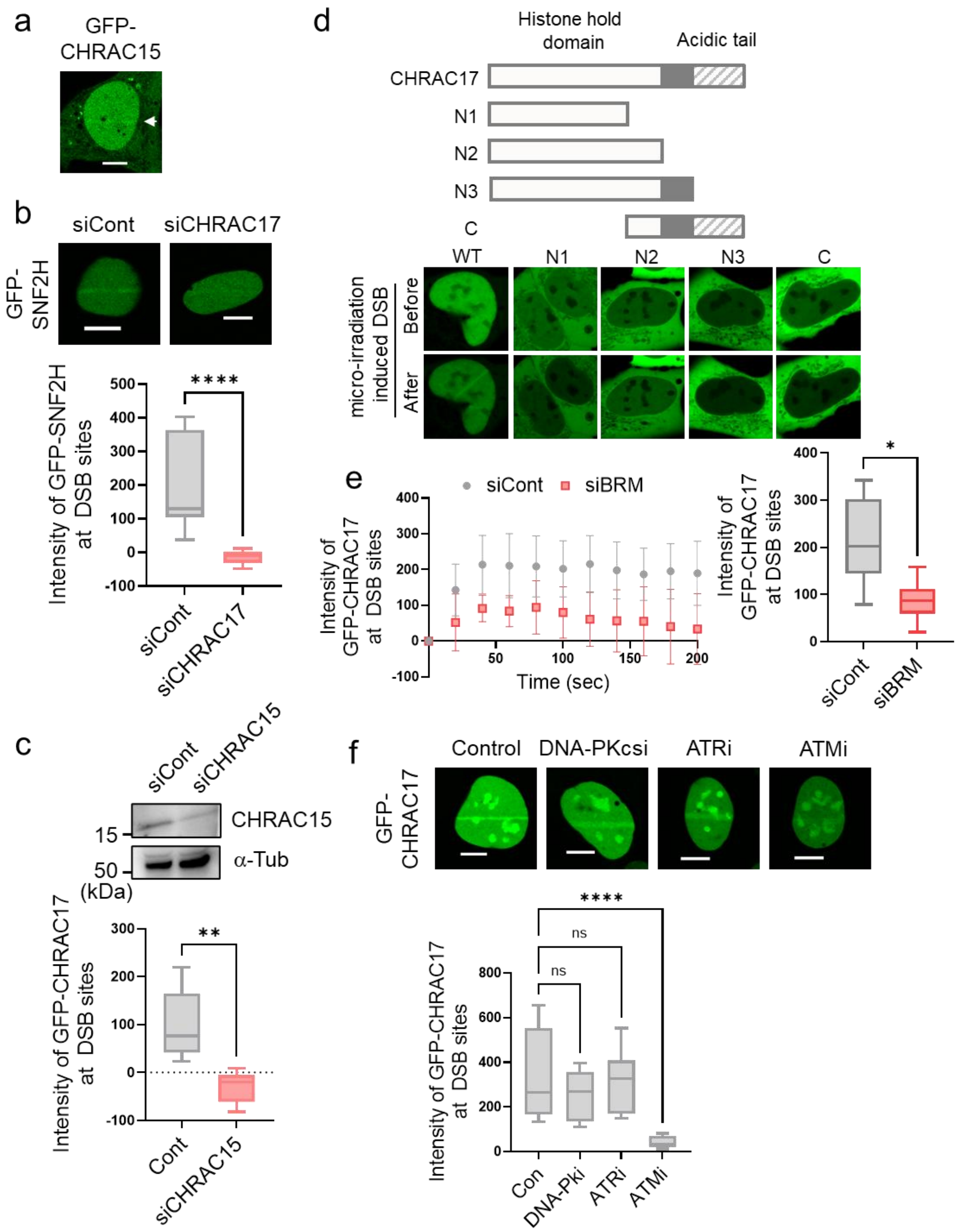

Supplementary Fig. S1

CHRAC17 is recruited at DNA double-strand breaks (DSBs)

(a) Recruitment of CHRAC15 at DSB sites. U2OS cells expressing GFP-CHRAC15 were subjected to laser micro-irradiation, and CHRAC15 accumulation at laser-micro-

irradiated sites was analyzed. Representative image of GFP-CHRAC15 at 200 s after micro-irradiation.

(b) CHRAC17 knockdown reduced accumulation of SNF2H at DSB sites. U2OS cells expressing GFP-SNF2H were subjected to laser micro-irradiation, and SNF2H accumulation at irradiated sites was analyzed. Intensity of GFP-SNF2H in U2OS cells at 200 s after laser-micro-irradiation.

(c) CHRAC15 knockdown reduced accumulation of CHRAC17 at DSB sites. H1299 cells expressing GFP-CHRAC17 were subjected to laser micro-irradiation, and CHRAC17 accumulation at irradiated sites was analyzed. Intensity of GFP-CHRAC17 in U2OS cells at 200 s after laser-micro-irradiation.

(d) Schematic of CHRAC17 deletion mutants. Representative images show that GFP-CHRAC17 deletion mutants do not localize to nuclei and do not accumulate at DSB sites. H1299 cells expressing GFP-CHRAC17 deletion mutants were subjected to laser micro-irradiation, and CHRAC17 accumulation at irradiated sites was analyzed.

(e) CHRAC17 accumulation at DSB sites is reduced by BRM knockdown. H1299 cells expressing GFP-CHRAC17 were subjected to laser micro-irradiation, and real-time accumulation at irradiated sites was monitored.

(f) ATM signaling promotes CHRAC17 recruitment to DSB sites. U2OS cells expressing GFP-CHRAC17 were treated with a DNA-PKcs inhibitor (NU7026), an ATR inhibitor (VE-821), or an ATM inhibitor (KU-55933), then subjected to laser micro-irradiation and imaged in real time. Intensity of GFP-CHRAC17 at 200 s after micro-irradiation. Quantification of real-time signal intensity at laser-micro-irradiated sites was performed using FLUOVIEW software (Olympus).

All statistical analyses were performed using GraphPad Prism. Significance was defined as ns (not significant) and $P < 0.05$ (*$P < 0.05$, **$P < 0.01$, ***$P < 0.001$, ****$P < 0.0001$; one-way ANOVA). Scale bar: 10 μm.

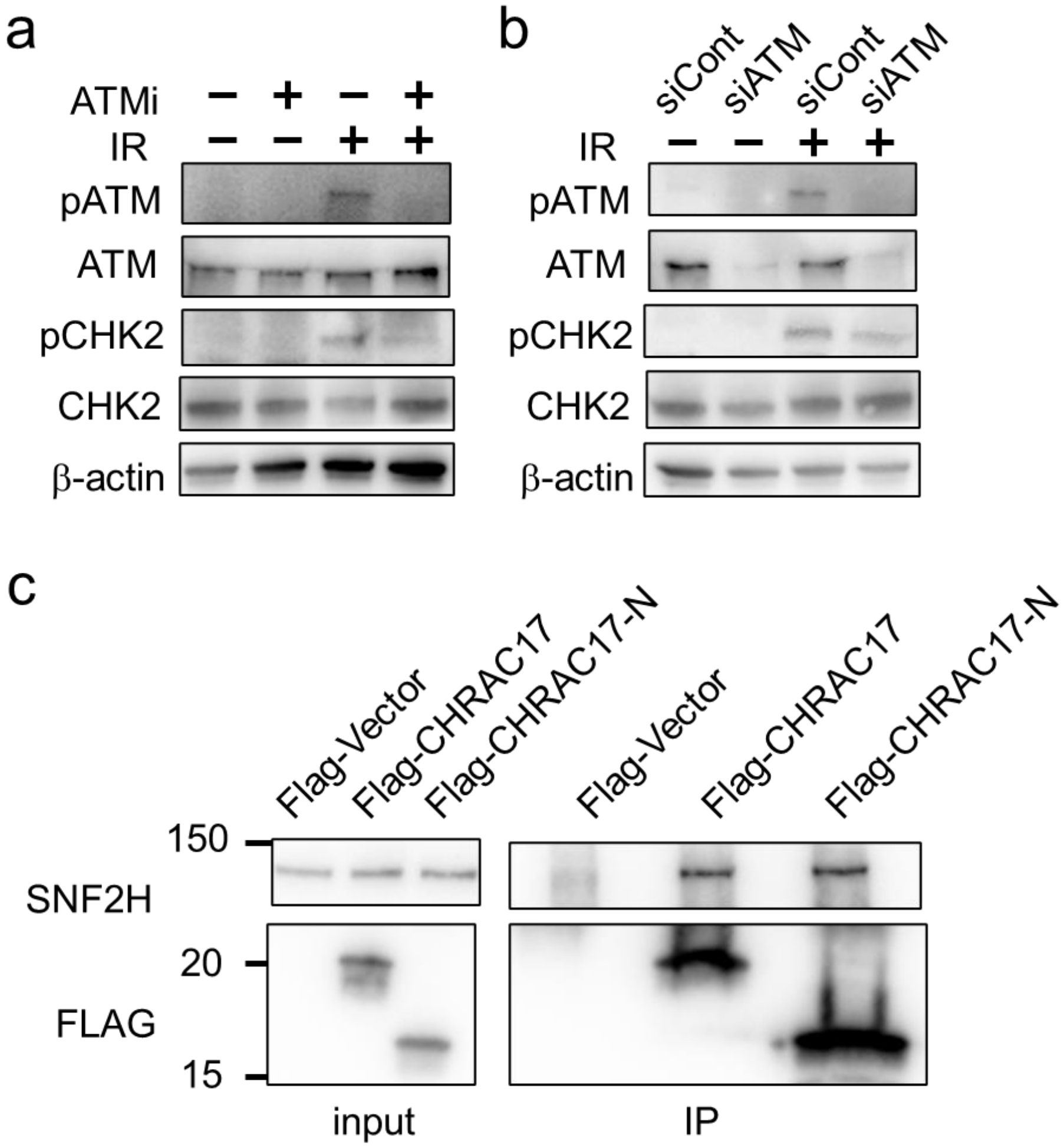

Supplementary Fig. S2

ATM inhibition reduces ATM and CHK2 phosphorylation following DSB induction, and CHRAC17-WT and CHRAC17-N interact with SNF2H.

(a) ATM inhibition reduced phosphorylation of ATM and CHK2 following DSB induction. U2OS cells were treated with an ATM inhibitor (KU-55933) and then exposed to ionizing radiation (IR). Whole-cell extracts were analyzed by Western blot with the indicated antibodies.

(b) ATM knockdown reduced phosphorylation of ATM and CHK2 following DSB induction. U2OS cells were transfected with siRNA targeting ATM and then exposed to IR. Whole-cell extracts were analyzed by Western blot with the indicated antibodies.

(c) CHRAC17-WT and CHRAC17-N interact with SNF2H. HEK293 cells expressing FLAG-tagged CHRAC17-WT or FLAG-tagged CHRAC17-N were subjected to

immunoprecipitation using an anti-FLAG antibody, followed by Western blot analysis with the indicated antibodies.

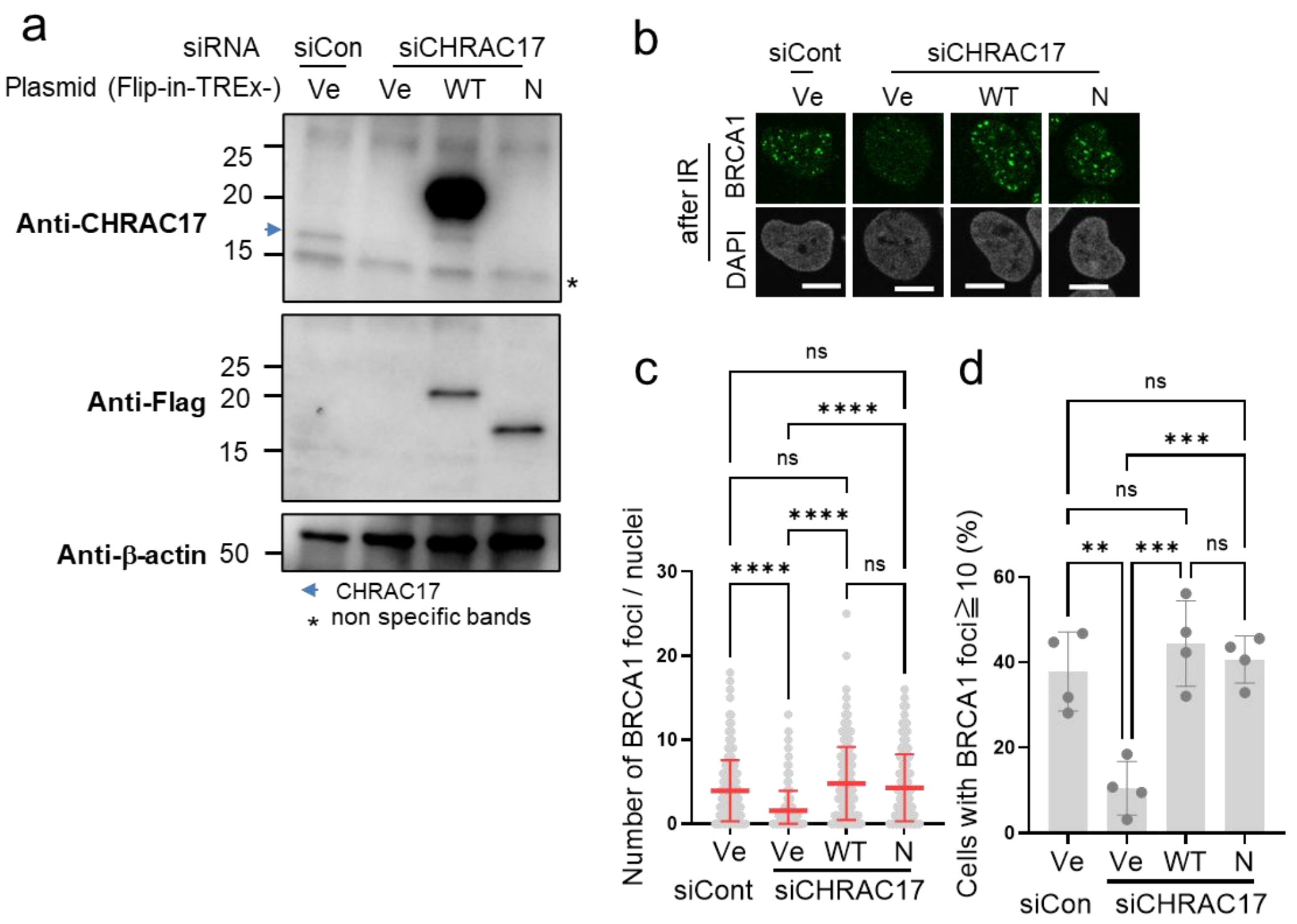

Supplementary Fig. S3

CHRAC17-WT and CHRAC17-N promote BRCA1 recruitment to DSB sites.

(a) Western blot analysis of HEK293 Flp-In T-REx cells expressing vector (Ve), FLAG-CHRAC17-WT (WT), and FLAG-CHRAC17-N (N), and endogenous CHRAC17, following transfection with siRNAs targeting the untranslated region of CHRAC17 (siCHRAC17-UTR).

(b-d) CHRAC17-N promotes BRCA1 recruitment to DSB sites. HEK293 T-REx cells expressing CHRAC17-N were treated with or without 2 Gy IR. After 2 h, cells were fixed and immunostained with anti-BRCA1 antibody. (b) Representative images. (c) Number of BRCA1 foci. (d) Percentage of cells with ≧10 BRCA1 foci. Data are presented as mean ± standard deviation from three technical replicates across two independent biological replicates.

All statistical analyses were performed using GraphPad Prism. Significance was defined as ns (not significant) and $P < 0.05$ (*$P < 0.05$, **$P < 0.01$, ***$P < 0.001$, ****$P < 0.0001$; one-way ANOVA). Scale bar: 10 µm.

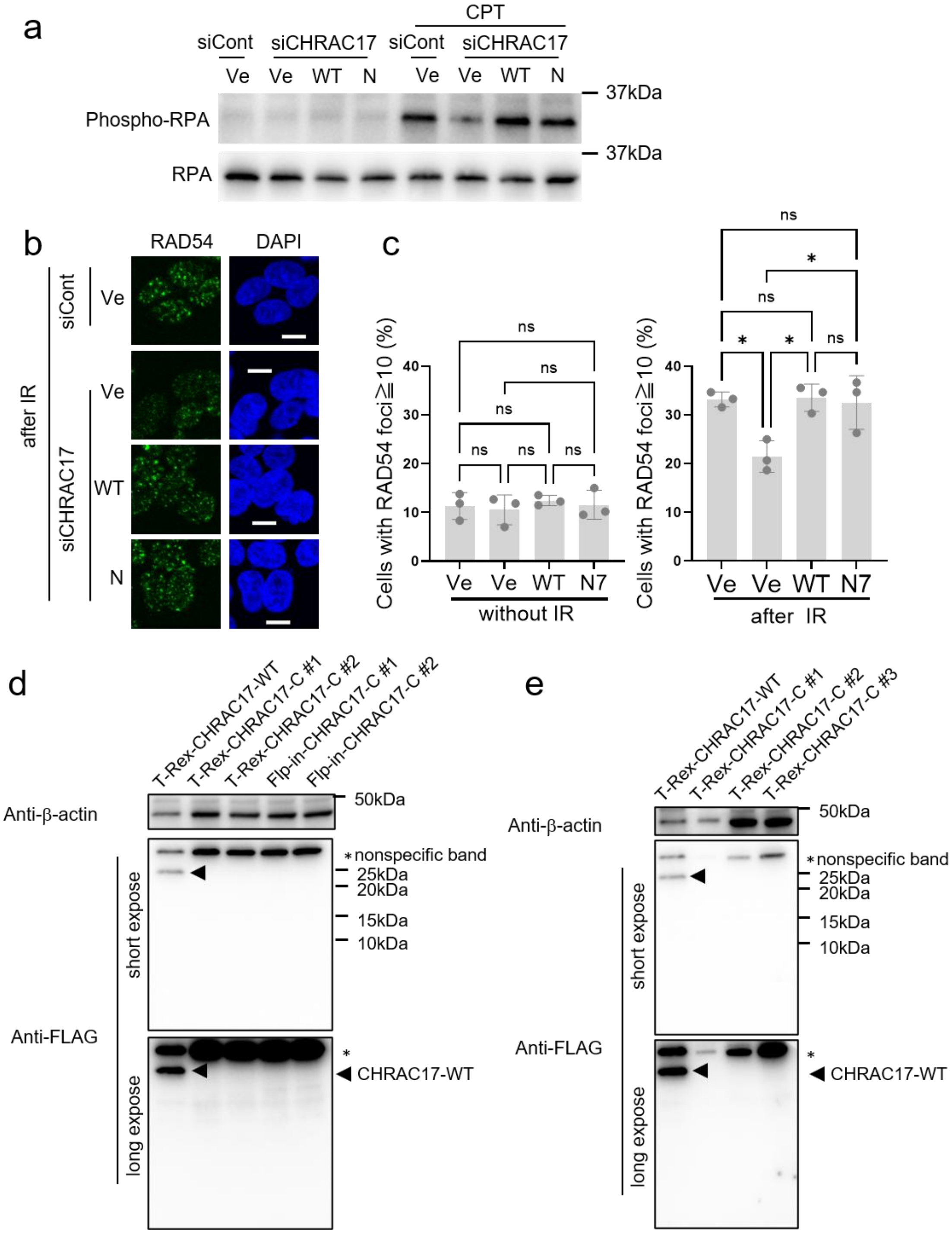

Supplementary Figure S4

CHRAC17-WT and CHRAC17-N promote DNA end resection and RAF54 recruitment to DSB sites.

(a) CHRAC17-WT and CHRAC17-N promote DNA end resection following

camptothecin (CPT) treatment. HEK293 T-REx cells expressing FLAG-vector (Ve), FLAG-CHRAC17-WT (WT), or FLAG-CHRAC17-N (N) were transfected with siRNA targeting the untranslated region of CHRAC17 (siCHRAC17-UTR) and treated with 1 μM CPT for 2 h. Whole-cell extracts were analyzed by Western blot with the indicated antibodies..

(b-c) CHRAC17-N promotes RAD54 recruitment to DSB sites. HEK293 T-REx cells expressing vector (Ve), CHRAC17-WT (WT), or CHRAC17-N (N) were transfected with siCHRAC17-UTR and treated with or without 2 Gy IR. After 2 h, cells were fixed and immunostained with anti-RAD54 antibody. (b) Representative images. (c) Percentage of cells with ≧10 RAD54 foci. Data are presented as mean ± standard deviation from three independent biological replicates.

All statistical analyses were performed using GraphPad Prism. Significance was defined as ns (not significant) and $P < 0.05$ (*$P < 0.05$, **$P < 0.01$, ***$P < 0.001$, ****$P < 0.0001$; one-way ANOVA). Scale bar: 10 μm.

(d) Generation of T-REx– and Flp-In T-REx–CHRAC17 C-terminal mutant expression constructs. Two independent CHRAC17 C-terminal mutant plasmids were generated and verified their sequences, and subsequently transfected into T-REx 293 cells. Protein expression was confirmed by Western blot analysis.

(e) Attempts to establish stable T-REx cell lines expressing the CHRAC17 C-terminal mutant. Stable T-REx cell lines were generated on three separate occasions. Protein expression was confirmed by Western blot analysis.

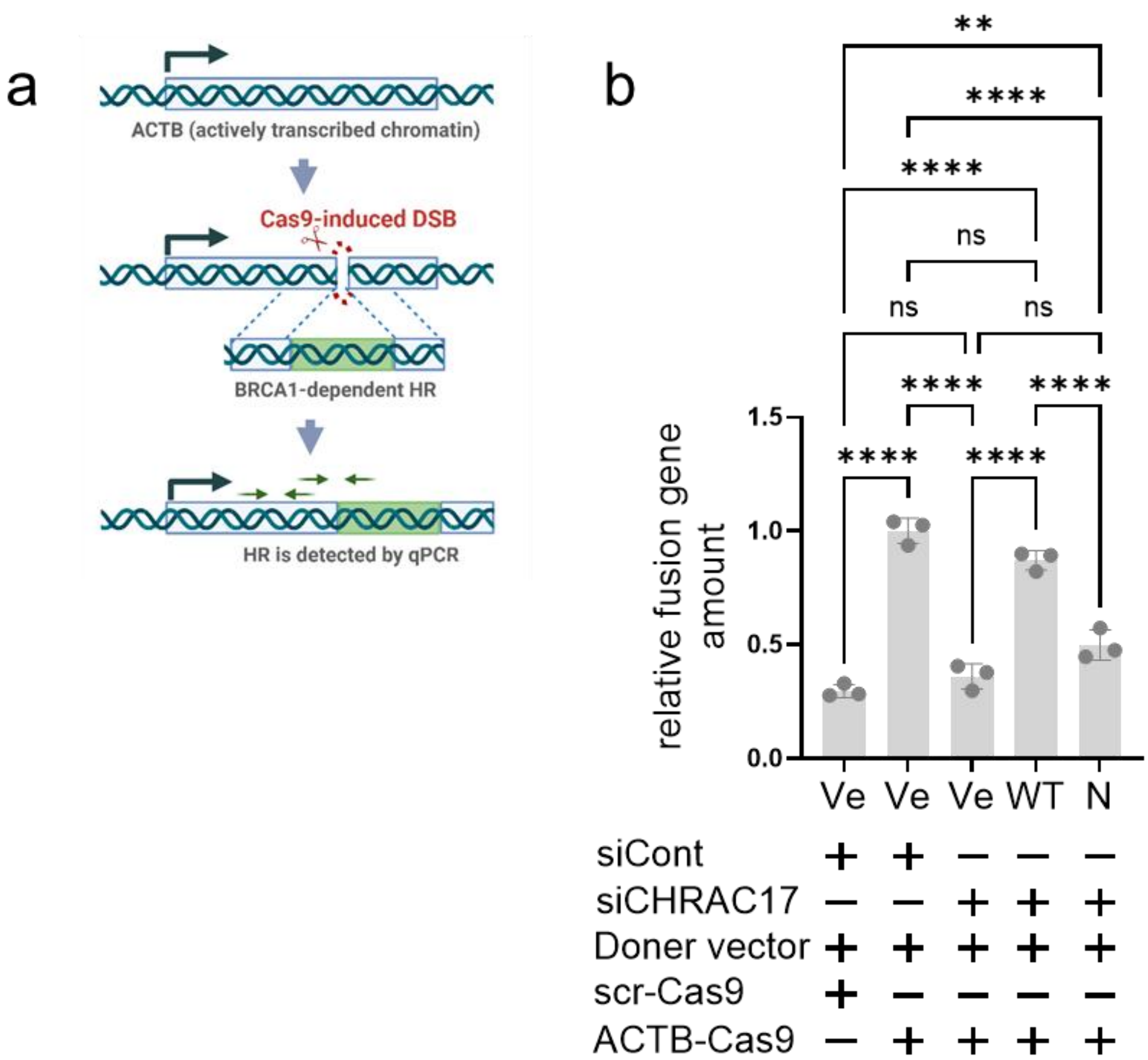

Supplementary Figure S5

CHRAC17-WT, but not CHRAC17-N, promotes BRCA1- and RAD51-mediated HR.

(a) A site-specific homologous recombination assay was performed in which a defined DSB is induced by CRISPR/Cas9, and successful BRCA1- and RAD51-mediated HR is quantified by real-time PCR using locus-specific primers 20. This assay directly measures completion of HR via formation of the repair product.

(b) CHRAC17-N failed to support BRCA1- and RAD51-mediated homologous recombination, as assessed by a site-specific HR assay. HEK293 T-REx cells expressing vector (Ve), CHRAC17-WT (WT), or CHRAC17-N (N) were transfected with siRNA targeting the untranslated region of CHRAC17 (siCHRAC17-UTR), followed by the HR assay. BRCA1- and RAD51-mediated HR was quantified by real-time PCR using locus-specific primers, and relative fusion gene levels were calculated. Data are presented as

mean ± standard deviation from three independent biological replicates.
All statistical analyses were performed using GraphPad Prism. Significance was defined as ns (not significant) and $P < 0.05$ (*$P < 0.05$, **$P < 0.01$, ***$P < 0.001$, ****$P < 0.0001$; one-way ANOVA).

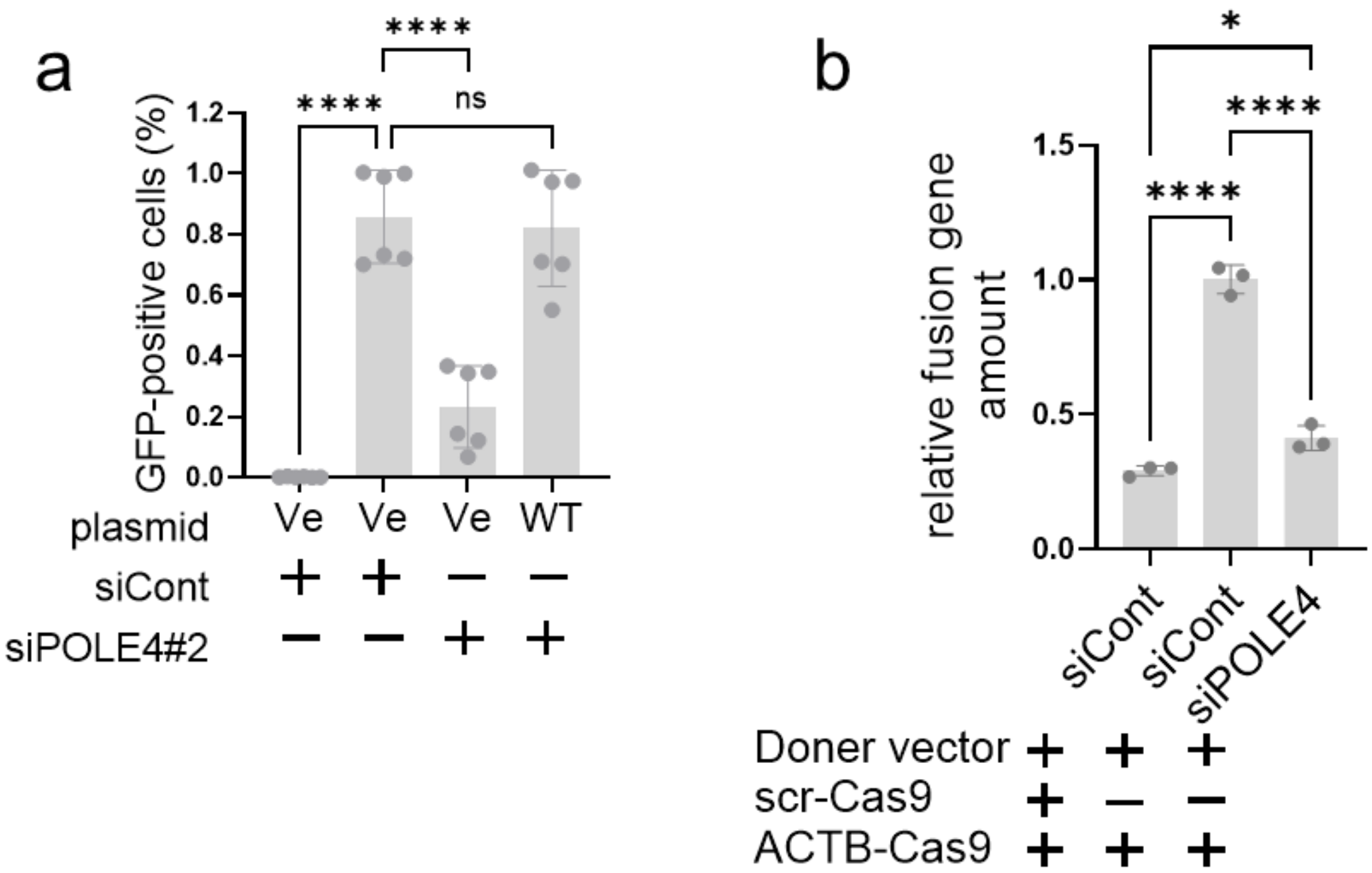

Supplementary Figure S6

POLE4 promotes HR activity and BRCA1- and RAD51-mediated HR

(a) POLE4 promotes HR activity as shown by a DR-GFP assay. POLE4 knockdown (POLE4 #2) reduced HR activity, and re-expression of POLE4 in cells treated with POLE4 #2, which targets the untranslated region of the POLE4 transcript, restored HR activity.

(b) POLE4 is required for completion of BRCA1- and RAD51-mediated homologous recombination, as assessed using a site-specific HR assay. HeLa cells were transfected with siRNA targeting POLE4, followed by the assay. BRCA1- and RAD51-mediated HR was quantified by real-time PCR using locus-specific primers, and relative fusion gene levels were calculated. Data are presented as mean ± standard deviation from three independent biological replicates.

All statistical analyses were performed using GraphPad Prism. Significance was defined as ns (not significant) and $P < 0.05$ (*$P < 0.05$, **$P < 0.01$, ***$P < 0.001$, ****$P < 0.0001$; one-way ANOVA).

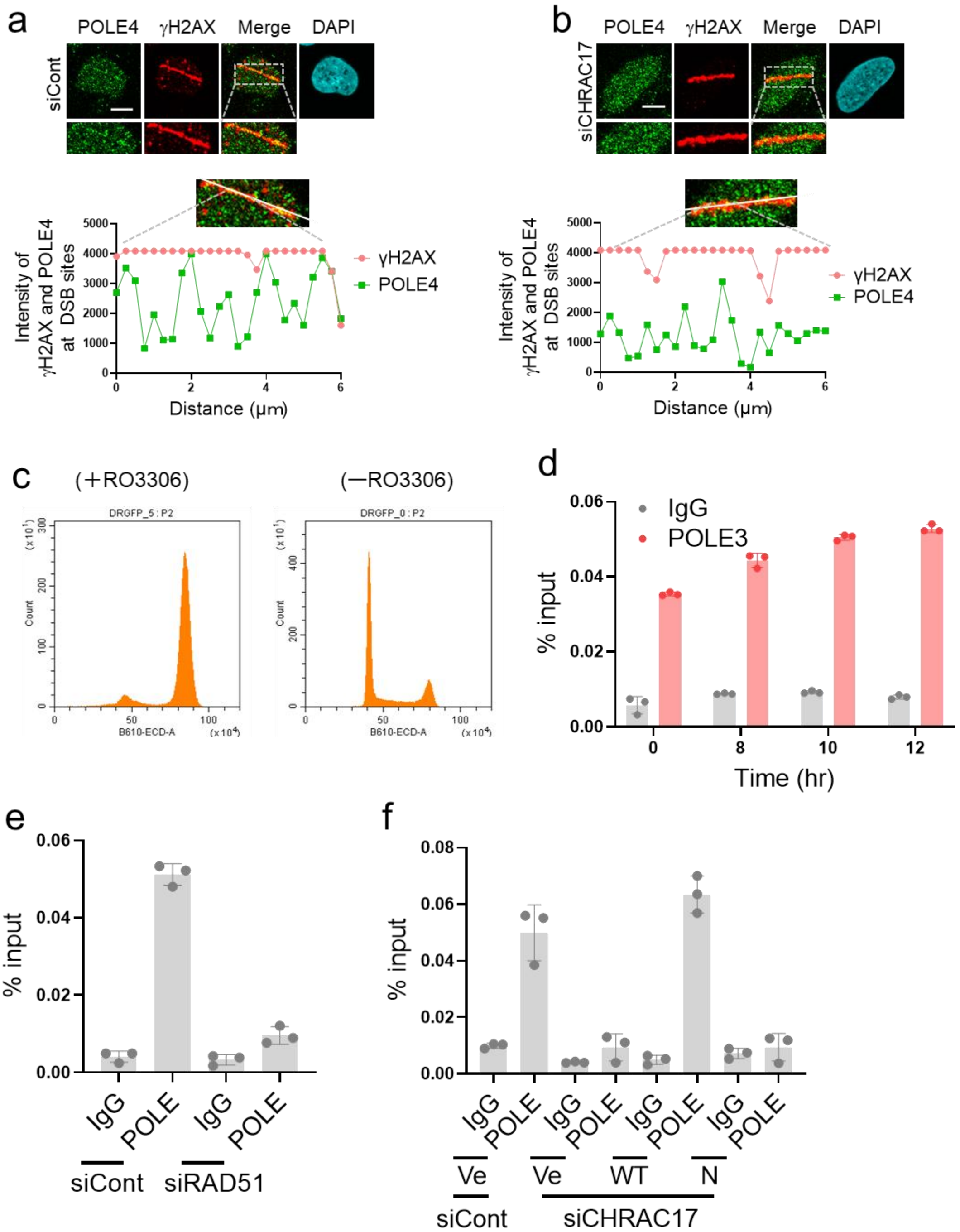

Supplementary Figure S7

POLE is recruited to DNA double-strand break (DSB) sites via RAD51 and CHRAC17

(a, b) POLE4 is recruited to DSB sites during the 4 h incubation following laser micro-irradiation. (a) Representative images of POLE4 recruitment in control cells. (b) CHRAC17-knockdown cells. (Lower panel) Quantification of signal intensity around

laser-micro-irradiated sites plotted against distance.

(c) DR-GFP HeLa cells were synchronized in G2 phase using RO3306. Cell-cycle status was analyzed by flow cytometry in cells treated with RO3306 (left) or untreated (right).

(d) ChIP analysis was performed using IgG (control) or anti-POLE3 antibody at 0, 8, 10, and 12 h after transfection of an I-SceI expression plasmid into G2-phase DR-GFP HeLa cells. Relative enrichment at each time point is shown.

(e) RAD51 promotes recruitment of POLE to DSB sites. ChIP analysis was performed using IgG (control) or anti-POLE2 antibody at 12 h after I-SceI transfection in G2-phase DR-GFP HeLa cells.

(f) Chromatin remodeling activity of CHRAC promotes recruitment of POLE to DSB sites. ChIP analysis was performed using IgG (control) or anti-POLE2 antibody at 12 h after I-SceI transfection in G2-phase DR-GFP HeLa cells.

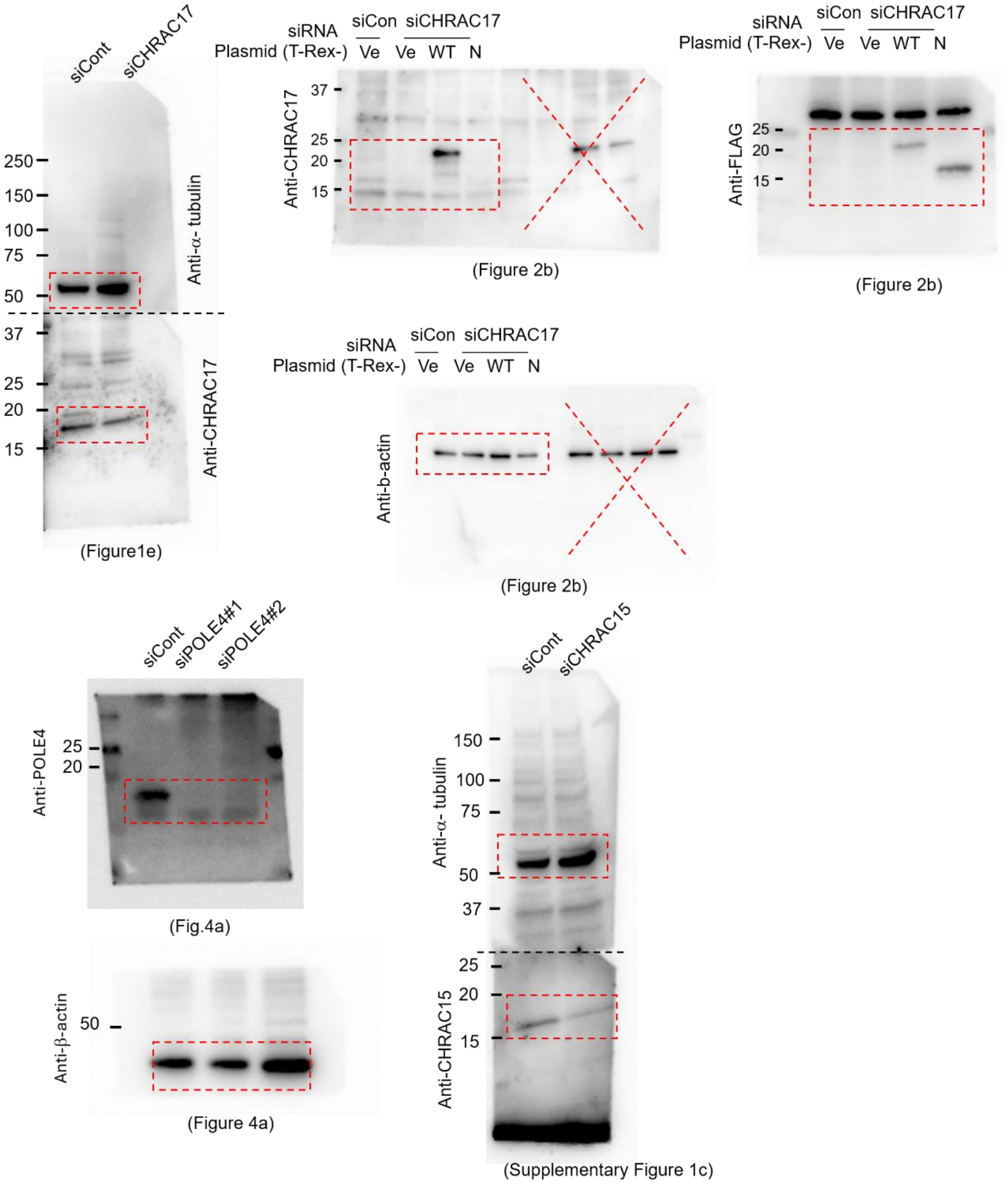

Supplementary Figure S8

Uncropped Western blot images corresponding the panels shown in Figure 1e, 2b, 4a and Supplementary Figure 1c. Rectangular boxes indicate the cropped regions used in the respective figure panels.

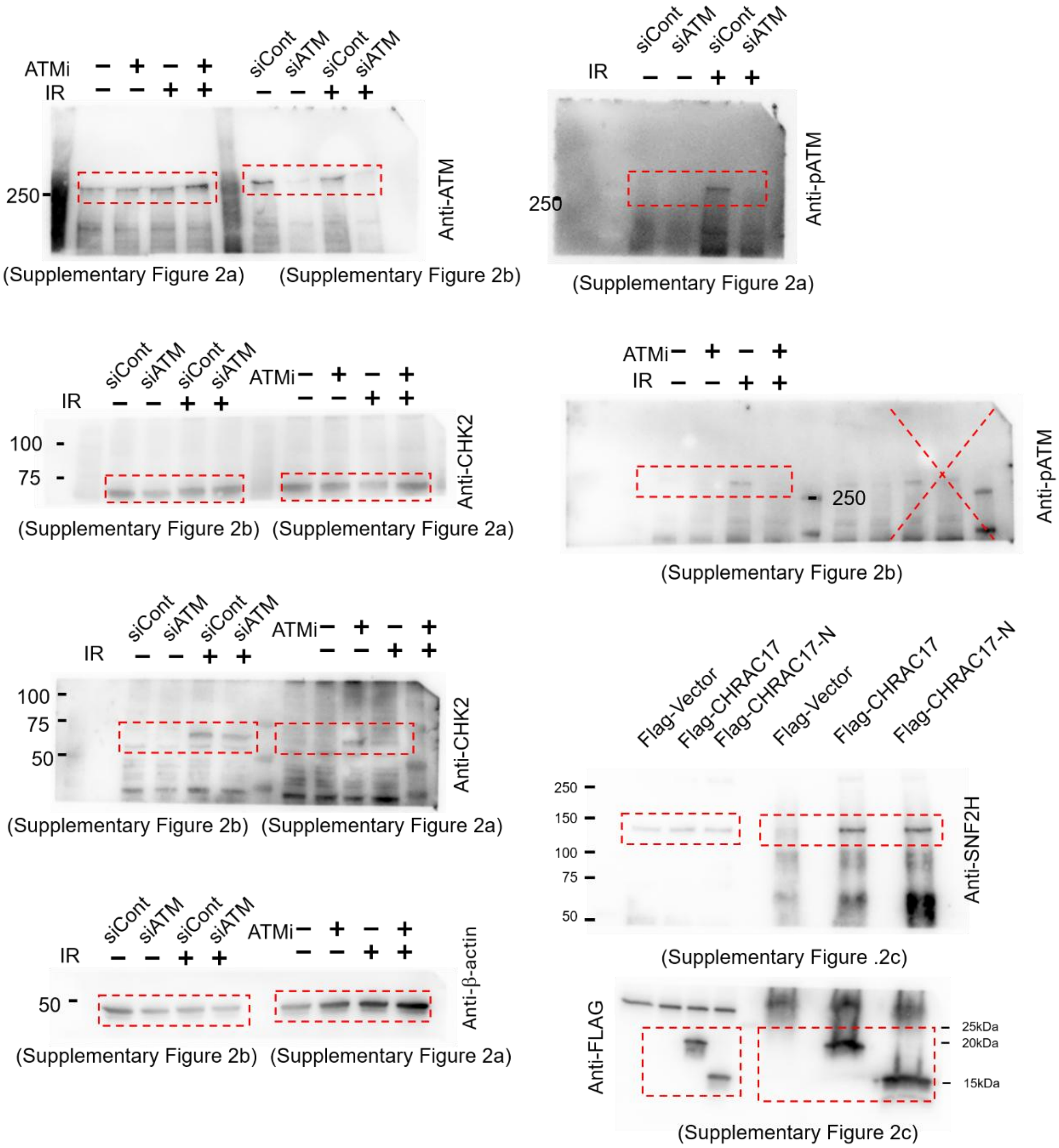

Supplementary Figure S9

Uncropped Western blot images corresponding the panels shown in Supplementary Figure 2a, 2b, and 2c. Rectangular boxes indicate the cropped regions used in the respective figure panels.

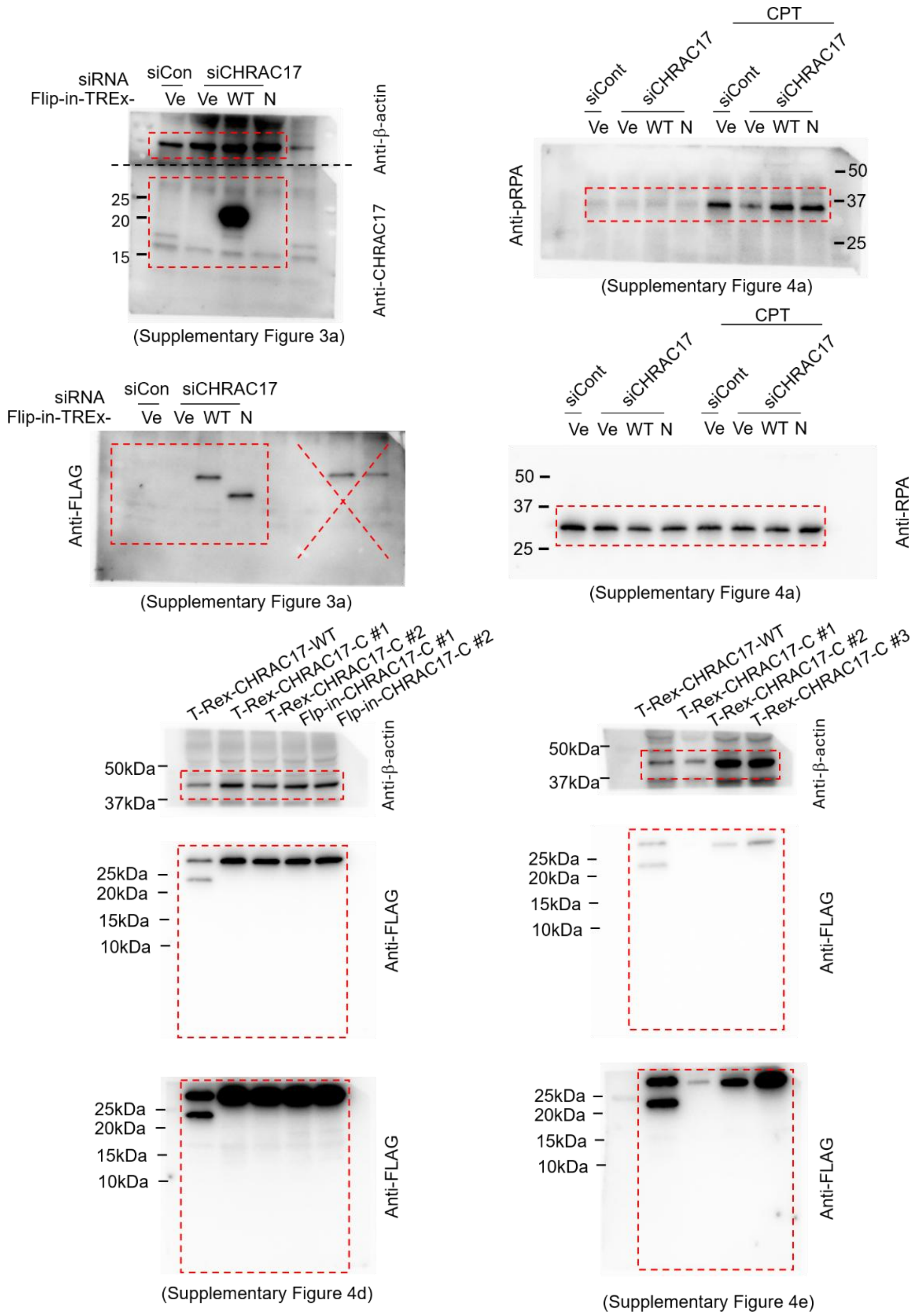

Supplementary Figure S10

Uncropped Western blot images corresponding the panels shown in Supplementary Figure 3a, 4a, 4d and 4e. Rectangular boxes indicate the cropped regions used in the respective figure panels.
