# Supplementary material for: ISWI/CHRAC orchestrates sequential chromatin-remodeling activities by mobilizing DNA polymerase ε for homologous recombination
Source: Commun Biol. 2026 Sep 15;9:1177. doi: 10.1038/s42003-026-10862-0 (PMC13578426; doi:10.1038/s42003-026-10862-0)
Supplement: Supplementary file 3 [file 42003_2026_10862_MOESM3_ESM.pdf]

Description of Additional Supplementary Files

File name: Supplementary Data

Description: The source data behind the graphs can be found in the Supplementary Data
