# Supplementary material for: ISWI/CHRAC orchestrates sequential chromatin-remodeling activities by mobilizing DNA polymerase ε for homologous recombination
Source: Commun Biol. 2026 Sep 15;9:1177. doi: 10.1038/s42003-026-10862-0 (PMC13578426; doi:10.1038/s42003-026-10862-0)
Supplement: Supplementary file 4 [file 42003_2026_10862_MOESM5_ESM.pdf]

nature portfolio

Corresponding author(s): Ayako Ui

Last updated by author(s): May, 28, 2026

Reporting Summary

Nature Portfolio wishes to improve the reproducibility of the work that we publish. This form provides structure for consistency and transparency in reporting. For further information on Nature Portfolio policies, see our Editorial Policies and the Editorial Policy Checklist.

Statistics

For all statistical analyses, confirm that the following items are present in the figure legend, table legend, main text, or Methods section.

n/a	Confirmed	
☐	☒	The exact sample size (*n*) for each experimental group/condition, given as a discrete number and unit of measurement
☐	☒	A statement on whether measurements were taken from distinct samples or whether the same sample was measured repeatedly
☐	☒	The statistical test(s) used AND whether they are one- or two-sided<br>*Only common tests should be described solely by name; describe more complex techniques in the Methods section.*
☒	☐	A description of all covariates tested
☐	☒	A description of any assumptions or corrections, such as tests of normality and adjustment for multiple comparisons
☐	☒	A full description of the statistical parameters including central tendency (e.g. means) or other basic estimates (e.g. regression coefficient) AND variation (e.g. standard deviation) or associated estimates of uncertainty (e.g. confidence intervals)
☐	☒	For null hypothesis testing, the test statistic (e.g. *F*, *t*, *r*) with confidence intervals, effect sizes, degrees of freedom and *P* value noted<br>*Give P values as exact values whenever suitable.*
☒	☐	For Bayesian analysis, information on the choice of priors and Markov chain Monte Carlo settings
☒	☐	For hierarchical and complex designs, identification of the appropriate level for tests and full reporting of outcomes
☒	☐	Estimates of effect sizes (e.g. Cohen's *d*, Pearson's *r*), indicating how they were calculated

Our web collection on statistics for biologists contains articles on many of the points above.

Software and code

Policy information about availability of computer code

Data collection	Data analyses were performed using GraphPad Prism 9.0 (GraphPad Software), ImageJ (NIH), FLUOVIEW (OLYMPUS)and CytExpert (Beckman Coulter Life Sciences)
Data analysis	Data were analyzed using ImageJ (NIH) and FLUOVIEW (OLYMPUS) for image quantification, GraphPad Prism version 9.0 (GraphPad Software) for statistical analysis and graph generation, and FCytExpert (Beckman Coulter Life Sciences) for flow cytometry data analysis. All analyses were performed according to standard procedures, and no custom code was used.

For manuscripts utilizing custom algorithms or software that are central to the research but not yet described in published literature, software must be made available to editors and reviewers. We strongly encourage code deposition in a community repository (e.g. GitHub). See the Nature Portfolio guidelines for submitting code & software for further information.

Data

Policy information about availability of data

All manuscripts must include a data availability statement. This statement should provide the following information, where applicable:

- Accession codes, unique identifiers, or web links for publicly available datasets
- A description of any restrictions on data availability
- For clinical datasets or third party data, please ensure that the statement adheres to our policy

The data and supporting materials are available within the article and from its online supplementary material

Research involving human participants, their data, or biological material

Policy information about studies with human participants or human data. See also policy information about sex, gender (identity/presentation), and sexual orientation and race, ethnicity and racism.

Reporting on sex and gender	n/a
Reporting on race, ethnicity, or other socially relevant groupings	n/a
Population characteristics	n/a
Recruitment	n/a
Ethics oversight	n/a

Note that full information on the approval of the study protocol must also be provided in the manuscript.

Field-specific reporting

Please select the one below that is the best fit for your research. If you are not sure, read the appropriate sections before making your selection.

☒ Life sciences ☐ Behavioural & social sciences ☐ Ecological, evolutionary & environmental sciences

For a reference copy of the document with all sections, see nature.com/documents/nr-reporting-summary-flat.pdf

Life sciences study design

All studies must disclose on these points even when the disclosure is negative.

Sample size	Sample sizes were determined based on previous similar studies and our experience with the variability of the assays.
Data exclusions	No data were excluded from the analyses. All obtained data points were included in the statistical analyses.
Replication	Each immunofluorescence and cell survival experiment was independently repeated at least two times with consistent results.
Randomization	Randomization was not applicable as the study involved cell-based experiments in which all samples were processed in parallel under identical conditions.
Blinding	Blinding was not applicable because all cell-based experiments were conducted under identical conditions, minimizing the potential for observer bias.

Reporting for specific materials, systems and methods

We require information from authors about some types of materials, experimental systems and methods used in many studies. Here, indicate whether each material, system or method listed is relevant to your study. If you are not sure if a list item applies to your research, read the appropriate section before selecting a response.

Materials & experimental systems

n/a	Involved in the study
☐	☒ Antibodies
☐	☒ Eukaryotic cell lines
☒	☐ Palaeontology and archaeology
☒	☐ Animals and other organisms
☒	☐ Clinical data
☒	☐ Dual use research of concern
☒	☐ Plants

Methods

n/a	Involved in the study
☒	☐ ChIP-seq
☐	☒ Flow cytometry
☒	☐ MRI-based neuroimaging

Antibodies

Antibodies used	γH2AX (05-636; MilliporeSigma), POLE2 (NBP1-87970; Novus), POLE3 (A301-245A; Thermo Fisher Scientific), b-actin (A5441; Sigma-Aldrich), POLE4 (PA5-97283; Thermo Fisher Scientific), BRCA1 (sc-6954; Santa Cruz Biotechnology), FLAG (F3165; Sigma-Aldrich), ATM (27156-1-AP; Proteintech), phosphorylated ATM (#5883; Cell signaling), ATM (27156-1-AP; Proteintech), phosphorylated CHK2

(#2348; Cell signaling), CHK2 (1395-4-1-AP; Proteintech), phosphorylated RPA (A300-245A; Cell signaling), RPA (10412-AP; Proteintech)

Validation: Each primary antibody was verified based on the manufacturer's official website.

Eukaryotic cell lines

Policy information about cell lines and Sex and Gender in Research

Cell line source(s)	NCI-H1299, U2OS , DR-GFP HeLa, Flp-In™T-REx™ 293
Authentication	Cell lines were obtained from authenticated sources (ATCC and Thermo Fisher Scientific). Authentication was performed by the supplier prior to distribution.
Mycoplasma contamination	cell lines were not tested for mycoplasma contamination
Commonly misidentified lines (See ICLAC register)	no

Plants

Seed stocks	no
Novel plant genotypes	no
Authentication	no

Flow Cytometry

Plots

Confirm that:

☒ The axis labels state the marker and fluorochrome used (e.g. CD4-FITC).

☒ The axis scales are clearly visible. Include numbers along axes only for bottom left plot of group (a 'group' is an analysis of identical markers).

☒ All plots are contour plots with outliers or pseudocolor plots.

☒ A numerical value for number of cells or percentage (with statistics) is provided.

Methodology

Sample preparation	After the cells were fixed with ethanol for 4 hours at 4?, they were washed with PBS. The cells were then incubated with 1 mL of RNase A (0.25 mg/mL) for 30 minutes at 37?. After washing again with PBS, the cells were stained with propidium iodide for 30 minutes at 4?and analyzed cell cycle by flow cytometry.
Instrument	CytoFLEX LX
Software	CytExpert
Cell population abundance	The abundance of each cell population (G1, S, and G2/M phases) was quantified using flow cytometry.
Gating strategy	Cells were gated based on forward and side scatter to exclude debris and doublets. Cell cycle phases (G1, S, and G2/M) were determined using propidium iodide staining and analyzed according to DNA content.

☒ Tick this box to confirm that a figure exemplifying the gating strategy is provided in the Supplementary Information.
